# Essential functions of RNA helicase Vasa in maintaining germline stem cells and piRNA-guided *Stellate* silencing in *Drosophila* spermatogenesis

**DOI:** 10.3389/fcell.2024.1450227

**Published:** 2024-08-09

**Authors:** Vladimir E. Adashev, Alexei A. Kotov, Sergei S. Bazylev, Ilia A. Kombarov, Oxana M. Olenkina, Aleksei S. Shatskikh, Ludmila V. Olenina

**Affiliations:** ^1^ Laboratory of Functional Genomics, Koltzov Institute of Developmental Biology, Russian Academy of Sciences, Moscow, Russia; ^2^ Department of Molecular Mechanisms for Realization of Genetic Information, National Research Centre Kurchatov Institute, Moscow, Russia

**Keywords:** piRNA pathway, germline stem cells, DEAD-box RNA helicase, spermatogenesis, male fertility

## Abstract

DEAD-box RNA helicase Vasa is required for gonad development and fertility in multiple animals. Vasa is implicated in many crucial aspects of *Drosophila* oogenesis, including translation regulation, primordial germ cell specification, piRNA silencing of transposable elements, and maintenance of germline stem cells (GSCs). However, data about Vasa functions in *Drosophila* spermatogenesis remain controversial. Here we showed that loss-of-function *vasa* mutations led to failures of GSC maintenance in the testes, a severe loss of total germ cell content, and a cessation of male fertility over time. Defects in GSC maintenance in *vasa* mutant testes were not associated with an increasing frequency of programmed cell death, indicating that a premature loss of GSCs occurred *via* entering differentiation. We found that Vasa is implicated in the positive regulation of *rhino* expression both in the testes and ovaries. The introduction of a transgene copy of *rhino*, encoding a nuclear component of piRNA pathway machinery, in *vasa* mutant background allowed us to restore premeiotic stages of spermatogenesis, including the maintenance of GSCs and the development of spermatogonia and spermatocytes. However, piRNA-guided repression of *Stellate* genes in spermatocytes of *vasa* mutant testes with additional *rhino* copy was not restored, and male fertility was disrupted. Our study uncovered a novel mechanistic link involving Vasa and Rhino in a regulatory network that mediates GSC maintenance but is dispensable for the perfect biogenesis of *Su(Ste)* piRNAs in testes. Thus, we have shown that Vasa functions in spermatogenesis are essential at two distinct developmental stages: in GSCs for their maintenance and in spermatocytes for piRNA-mediated silencing of *Stellate* genes.

## Introduction

DEAD-box RNA helicase Vasa (also known as DDX4) is a conserved protein involved in the germline specification and gametogenesis of multiple Metazoa species (Roussell and Bennett, 1993; Lasko, 2013; Hartung et al., 2014; Adashev et al., 2023). As all DEAD-box family proteins, Vasa possesses a highly conserved core helicase region that contains at least 12 conservative motifs distributed between two RecA-like domains, called the N-terminal DEAD-like helicase domain (DEXDc) and the C-terminal helicase superfamily domain (HELICc). These core domains bind both ATP and RNA molecules and ensure RNA helicase and ATPase activity (Linder and Jankowsky, 2011; Wang et al., 2015). The biochemical activities of DEAD-box helicases include the local RNA duplex unwinding, the remodeling of ribonucleoprotein complexes, the facilitation of RNA renaturation, and RNA clamping (Linder and Jankowsky, 2011; Kotov et al., 2014). DEAD-box helicases are known to contribute to all aspects of intracellular RNA metabolism.

Initially, gene *vasa* has been discovered in fruit flies from the screens for maternal-effect recessive lethal mutations (Schüpbach and Wieschaus, 1986; Schüpbach and Wieschaus, 1989). Functions of the RNA helicase Vasa in *Drosophila* are essential for primordial germ cell (PCG) specification and embryonic development, as well as for the progression of oogenesis (Lasko and Ashburner, 1990; Styhler et al., 1998; Durdevic et al., 2018; Durdevic and Ephrussi, 2019; Hansen and Pelegri, 2021). A deficiency of *vasa* contributes to multiple defects of oogenesis and leads to female sterility in *Drosophila* (Carrera et al., 2000; Johnstone and Lasko, 2004; Liu et al., 2009; Durdevic et al., 2018; Durdevic and Ephrussi, 2019). Only a small number of oocytes in *vasa* mutant females reach maturation, while the bulk of them undergo premature degeneration in the early oogenesis stages. In the ovaries of *vasa* mutants, the atrophy of germarium and a loss of early germ cells, including germline stem cells (GSCs) and cystoblasts, are often detected (Lasko and Ashburner, 1990; Styhler et al., 1998). However, the molecular functions of Vasa in oogenesis remain poorly understood.

In the *Drosophila* germline, Vasa is enriched in *nuage*, the perinuclear granules of ovarian nurse cells and premeiotic germ cells in the testes (Snee and Macdonald, 2004; Lim and Kai, 2007; Malone et al., 2009; Kibanov et al., 2011). Granules *nuage* are intracellular membraneless organelles that are assembled as liquid biomolecular condensates (Nott et al., 2015; So et al., 2021). They are known as the sites of posttranscriptional piRNA biogenesis and posttranscriptional piRNA-mediated silencing of selfish genomic elements, including transposons, genome repeats, and protein-coding genes (Lim and Kai, 2007; Malone et al., 2009; Kibanov et al., 2011; Webster et al., 2015). Vasa is a basic hierarchical structural component of *nuage* that recruits ARGONAUTE/PIWI family endonucleases Aubergine (Aub) and AGO3 in *nuage* (Lim and Kai, 2007; Kibanov et al., 2011; Yamazaki et al., 2023). Vasa appears to participate in the transfer of long piRNA precursors from the nucleus to *nuage* granules (Zhang et al., 2012) and contribute to the piRNA amplification ping-pong cycle carried out by Aub and AGO3 proteins for the tuning of target silencing process (Brennecke et al., 2007; Gunawardane et al., 2007; Malone et al., 2009; Xiol et al., 2014; Webster et al., 2015). Vasa helicase activity is required for the piRNA-mediated silencing of target transcripts (Durdevic and Ephrussi, 2019). Loss-of-function *vasa* mutations lead to a failure of *nuage* assembly. It causes the cytoplasmic distribution of Aub, AGO3, and other components of the piRNA machinery and the disruption of piRNA-mediated silencing in the germline (Vagin et al., 2006; Lim and Kai, 2007; Malone et al., 2009; Kibanov et al., 2011; Kotov et al., 2014). In *vasa* mutant females, a lack of piRNA production in ovarian nurse cells leads to the mobilization of multiple transposable elements (TEs) (Vagin et al., 2004; Malone et al., 2009; Czech et al., 2013). A transient loss of Vasa expression only between oogenesis stages 2 and 6 causes the accumulation of TE transcripts in ovarian nurse cells (Durdevic et al., 2018). High-level TE activation leads to the transportation of their transcripts in the developing oocyte and their integration into the oocyte genome (Wang et al., 2018). Insertions of TEs cause DNA double-strand breaks in various loci of the genome, causing a loss of chromatin integrity and replication stress. It can also trigger oogenesis arrest through activation of the checkpoint kinase 2 (Chk2) signaling cascade (Klattenhoff et al., 2007; Durdevic et al., 2018). Elevated level of TE activity can also result in vertical TE transfer from mothers to progeny, causing chromatin damage and mitotic arrest in early embryogenesis (Durdevic et al., 2018; Wang et al., 2018). Vasa is expressed in the germline throughout the whole fly oogenesis and is also enriched on the posterior pole of the developing oocyte in so-called pole granules or pole plasm that is essential for primordial germ cell specification in embryos (Hay et al., 1988; Lasko and Ashburner, 1990; Trcek and Lehmann, 2019).

Despite the fact that Vasa is expressed in both the ovarian and testis germline, data about its functions in spermatogenesis remain controversial. Fertility of males carrying *vasa* mutations leads to the proposal that *vasa* have no essential functions in spermatogenesis (Lasko and Ashburner, 1988; Lasko, 2013). In the testes of *vasa* mutant males, TE mobilization is less conspicuous, but protein-coding tandem *Stellates* genes become strongly derepressed in spermatocytes (Aravin et al., 2001; Vagin et al., 2006; Kibanov et al., 2011; Adashev et al., 2021), which leads to subsequent meiotic disorders and male fertility disturbances (Palumbo et al., 1994; Bozzetti et al., 1995). Thus, data about the involvement of Vasa in the piRNA pathway and *Stellate* gene silencing in testes clearly conflict with the above-mentioned point of view (Vagin et al., 2006; Kibanov et al., 2011). Since Vasa contributes to various intracellular processes in the germline, studying its functions is difficult. It remains unclear whether its functions in the piRNA pathway and in other developmental processes are mutually linked or relatively independent.

**FIGURE 1:**
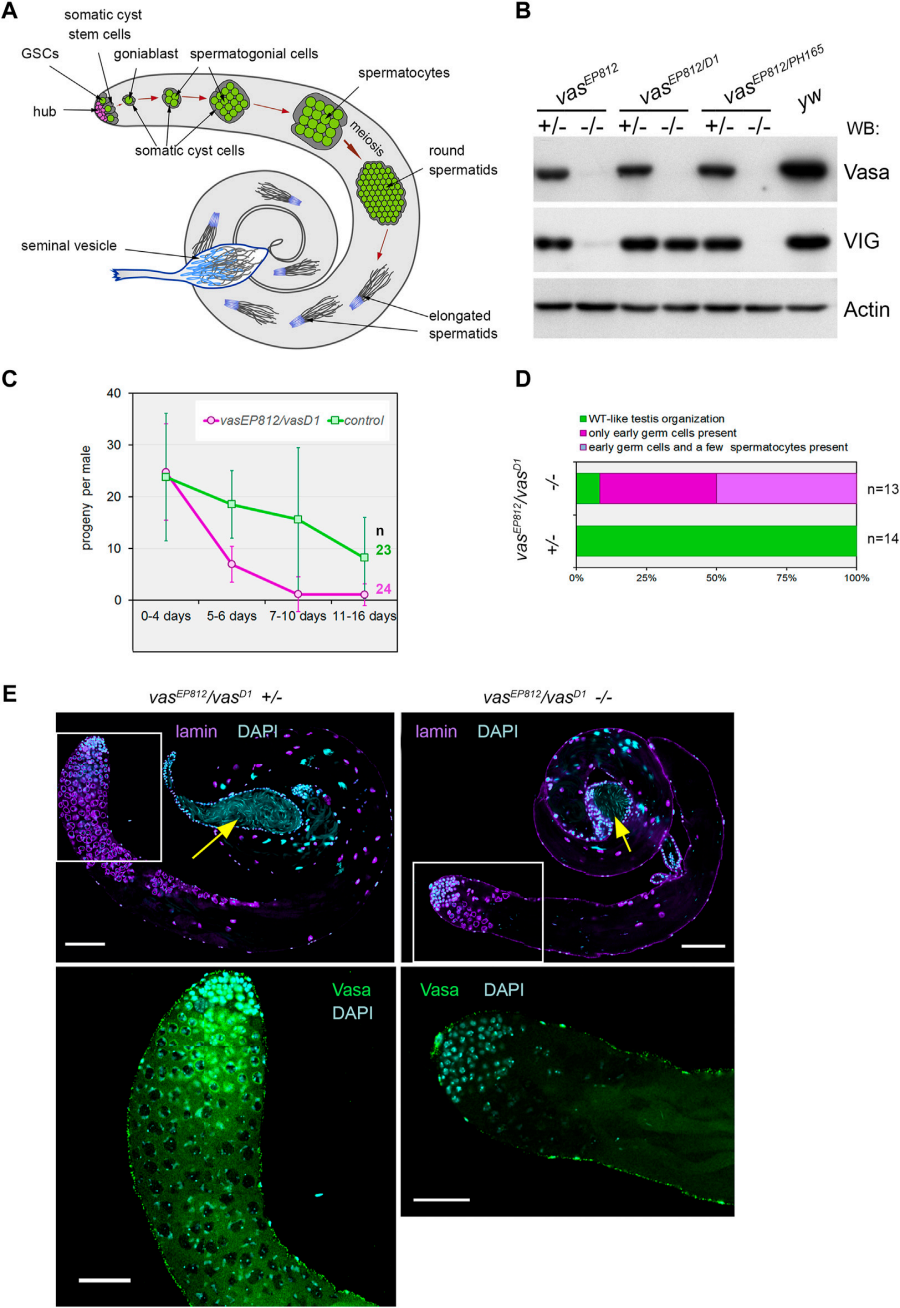
Vasa is required for male fertility and germ cell maintenance in the testes. **(A)** A scheme of *Drosophila* spermatogenesis. Germline stem cells (GSCs, green) are located adjacent to the hub (violet) at the apical testis tip and are encapsulated by two somatic cyst stem cells (CySCs, grey). The daughter of the GSC, the goniablast, undergoes four mitotic divisions to create cysts of 16 spermatogonia, overlapping two undivided somatic cyst cells. After that, the germ cells within the cyst switch to the spermatocyte program. Spermatocytes undergo extensive cell growth and then enter synchronous meiosis with the appearance of 64 haploid round spermatids. Spermatids elongate and move to the basal end of the testis. Mature individual spermatozoa enter the seminal vesicle and are stored there as mature sperm. **(B)** Western blot analysis of the testis lysates of *vasa* mutants. Note that the testes of *vas* ^*EP812*^ */vas* ^*D1*^ heteroallelic combination expressed VIG but not Vasa, whereas *vas* ^*EP812*^ */vas* ^*PH165*^ and *vas* ^*EP812*^ */vas* ^*EP812*^ mutations caused a loss of expression of both Vasa and VIG proteins. Anti-Actin antibodies were used as a loading control. **(C)** Fertility test of *vasa* mutant males with *vas* ^*EP812*^ */vas* ^*D1*^ heteroallelic combinations (violet lines) in comparison with their heterozygous siblings (green lines). The average offspring number of male per day with standard errors is presented for indicated time intervals after parent male eclosion. The number of examined males is indicated. **(D)** The bar diagram depicts the distribution of testis phenotypes of *vas* ^*EP812*^ */vas* ^*D1*^ mutants and control heterozygous males (3 days after eclosion): wild-type-like phenotype (green), only early germ cells (violet), early germ cells and a few spermatocytes (blue). The numbers of examined testes are indicated. **(E)** A loss of germ cells in the testes of *vas* ^*EP812*^ */vas* ^*D1*^ heteroallelic mutants. Left: testes of heterozygous control males (3 days after eclosion) exhibited a wild-type phenotype. Right: testes of *vas* ^*EP812*^ */vas* ^*D1*^ heteroallelic mutants (3 days after eclosion) rapidly lost germline content. Whole-mount fixed testes were immunostained with Vasa (green) and lamin (violet) antibodies, and chromatin was stained by DAPI (blue). Confocal slices with the apical tips of the testes oriented leftward are shown. Fragments in the white boxes of the top images are present in higher magnifications in the corresponding bottom images with Vasa and DAPI staining. Scale bars are 100 µm for the top images and 50 µm for the bottom ones. Yellow arrows indicate seminal vesicles containing mature sperm.

Adult males of *Drosophila* contain a pair of testes; each of them is represented by an elongated and twisted tube. In wild-type testes, Vasa-positive germline stem cells (GSCs) reside at the apical tip of the testis around a cohort of terminally differentiated somatic cells, representing a niche structure termed the hub (Figure 1A). The hub supports two adjacent stem cell populations with the aid of the secretion of signaling molecules: 9–12 germline stem cells (GSCs) and twice as many somatic cyst stem cells (CySCs). The self-renewal division of a GSC provides a new GSC and a goniablast (spermatogonium cell). The division of CySC generates a self-renewing CySC and a cyst cell. A pair of cyst cells overlap a spermatogonium, forming the cyst, a functional unit of spermatogenesis. Spermatogonial cells inside the cyst undergo four rapid mitotic divisions with incomplete cytokinesis, finally providing a 16-cell syncytium of primary spermatocytes surrounded by two undivided flattened CyCs. Primary spermatocytes enter a long premeiotic interphase accompanied by extensive cell growth. After that, mature spermatocytes undergo synchronous meiosis with the appearance of 64 haploid round spermatids. Spermatids elongate and undergo the individualization process, move to the basal end of the testis, enter the seminal vesicle, and are stored there as mature sperm before copulation (Spradling et al., 2011; Greenspan et al., 2015) (Figure 1A).

Our study explored the molecular functions of Vasa in testes of *D. melanogaster*. Here we found that loss-of-function *vasa* mutations led to a rapid loss of GSC number in testes and a decrease in male fertility with aging. Defects in GSC maintenance in *vasa* mutant testes were not associated with an elevated frequency of premature cell death, indicating that a loss of GSCs occurred through entering differentiation without a self-renewal. According to our data, two main functions of RNA helicase Vasa in the testes of *Drosophila melanogaster* are carried out in distinct cohorts of germ cells: the maintenance of GSCs in early spermatogenesis and piRNA silencing of *Stellate* genes, ensuring correct passage through meiosis, in spermatocytes. Both of these functions are necessary for male fertility, underscoring the essential role of Vasa in fly spermatogenesis.

## Results

### Vasa is required for male fertility in *Drosopila*

We revealed that *vas* ^*EP812*^ */vas* ^*PH165*^ and *vas* ^*EP812*^ */vas* ^*EP812*^ mutations caused a loss of expression of both Vasa and VIG (a product of the *vasa intronic gene* also encoding the *vasa* locus) proteins in the testes, whereas *vas* ^*EP812*^ */vas* ^*D1*^ heteroallelic combination only disrupted Vasa expression but did not disrupt half-dose VIG expression (Figure 1B). The *vas* ^*D1*^ loss-of-function allele has been obtained using ethyl methanesulfonate mutagen with no changes in the coding region (Lasko and Ashburner, 1990). *vas* ^*D1*^ is not a null allele that appears to be associated with a disturbance of *vasa* transcriptional regulation (Lasko and Ashburner, 1990; Liang et al., 1994). Using males carrying loss-of-function *vasa* mutations, we performed male fertility tests over a long period of time (described in Materials and Methods). We found that young *vas* ^*EP812*^ */vas* ^*D1*^ mutant males (0–4 days after eclosion) give rise to adult offspring in crossing with *yw* females, providing a progeny number comparable to that of control heterozygous siblings (Figure 1C). However, already from the fifth day onwards, a drastic fertility reduction of mutant males compared to control heterozygous siblings was observed. In subsequent time intervals (7–10 days and 11–16 days), most *vas* ^*EP812*^ */vas* ^*D1*^ males produce no progeny (Figure 1C). Testing males with heteroallelic combinations *vas* ^*EP812*^ */vas* ^*PH165*^, we found that even though mutant males were fertile at a low level up to the twentieth day after eclosion, in subsequent time intervals their fertility declined to a very low level (Supplementary Figure S1A). Taken together, these results demonstrate that male reproductive capacity is substantially lowered in the absence of Vasa expression, indicating the indispensable role of Vasa in male fertility maintenance.

Further, we examined the morphology of the testes of *vas* ^*EP812*^ */vas* ^*D1*^ mutant males (3 days after eclosion). We found that whereas the testes of heterozygous siblings exhibited a wild-type distribution of premeiotic Vasa-positive germ cells, the *vas* ^*EP812*^ */vas* ^*D1*^ testes exhibited a significant loss of the total pool of germ cells in the testes (Figures 1D, E). However, seminal vesicles in both control and mutant young males were filled with mature sperm (Figure 1E, yellow arrows), supporting that the first waves of spermatogenesis in *vasa* mutant males proceeded correctly. We also observed a similar loss of germline content for other *vasa* mutant males, *vas* ^*EP812*^ */vas* ^*PH165*^ (Supplementary Figures S1B, C)*.* In sum, in *vasa* mutant males, a rapid decline in germ cell number in the testes is accompanied by reduced fertility.

### Defects of germline stem cells maintenance in the testes of *vasa* mutant males

**FIGURE 2:**
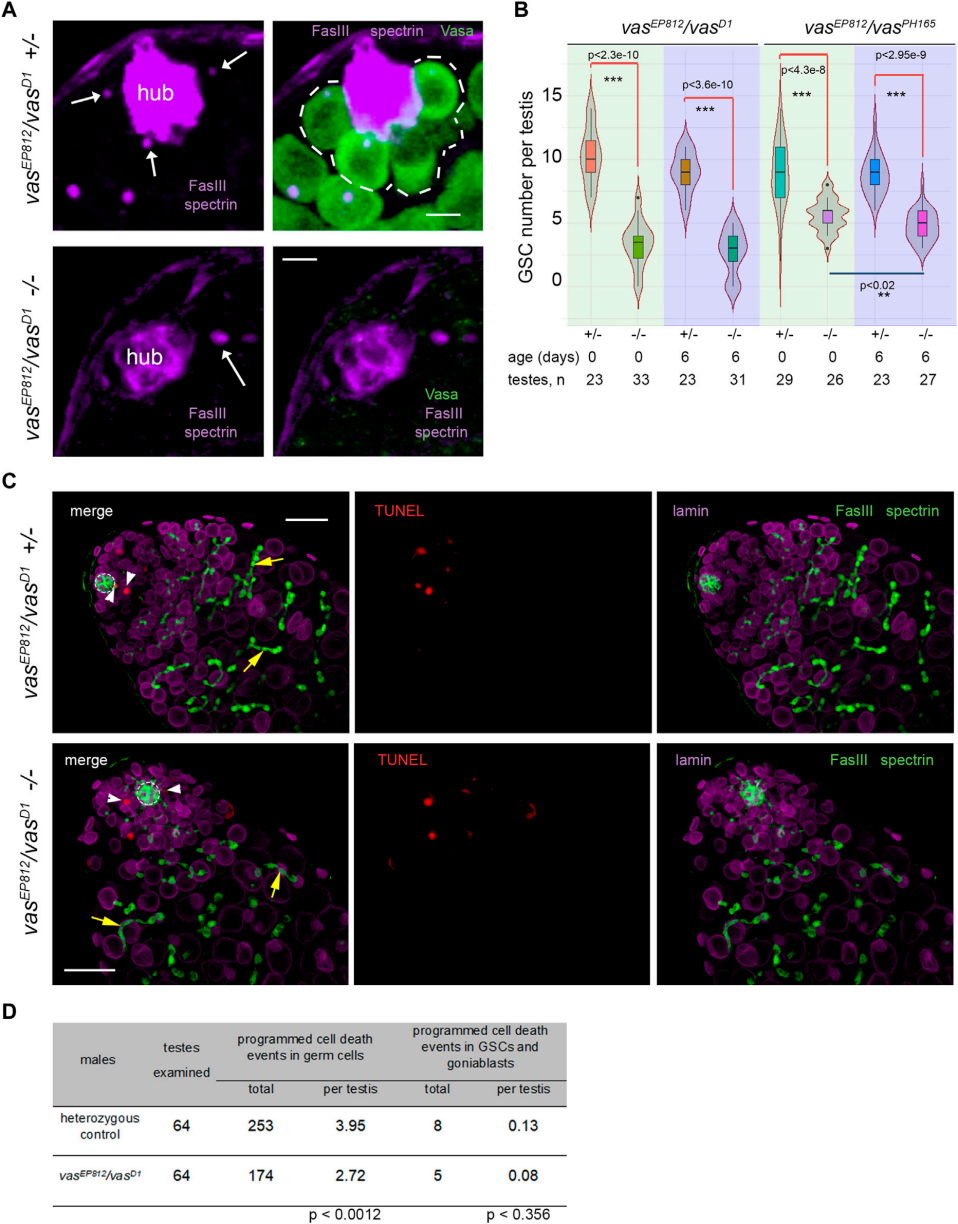
Analysis of the testes of *vasa* mutants. **(A)** GSCs in the testes of *vasa* mutants and control heterozygous siblings. Testes of mutant and heterozygous *vas* ^*EP812*^ */vas* ^*D1*^ control males (0 days) were immunostained with Vasa (green), Fasciclin III (violet, marker of hub), α-spectrin (violet) antibodies, and DAPI (blue) staining. White arrows indicate spectrosomes, dot-like GSC-specific organelles located near the hub. Single confocal slices of testis tip images are shown. Scale bars are 5 µm. **(B)** Loss of GSCs in the testes of *vas* ^*EP812*^ */vas* ^*D1*^and *vas* ^*EP812*^/*vas* ^*PH165*^ mutants. Violin plots present GSC number per testis in *vasa* mutant and heterozygous control testes of 0-day-old and 6-day-old males. The medians and quartiles of GSC number sets are marked. The number of testes counted for each genotype and the age of the analyzed males are shown at the bottom. *** Significant differences (from *p* < 4.3e-8 to *p* < 2.3e-10) are found by pairwise comparison of GSC numbers in the mutant and control testes of the same age (Wilcoxon/Mann-Whitney (U) test). ** Between the testes of 0- and 6-day *vas* ^*EP812*^ */vas* ^*PH165*^ mutant males, significant differences in GSC number were also found (*p* < 0.02). **(C)** TUNEL assay (red signals) with simultaneous immunostaining with antibodies to Fasciclin III (FasIII, hub marker, green), α-spectrin (marker spectrosomes and fusomes, green), and lamin (marker nuclear envelope, violet). Confocal slices with the apical tips of testes oriented leftward here and after are shown. The hubs are marked by white outlines. TUNEL signals in GSCs and goniablasts are indicated by white arrowheads. The fusomes are indicated by yellow arrows. Scale bars are 20 µm. **(D)** Analysis of programmed cell death events in mutant and control testes. Data are presented as counted TUNEL-positive signals and as the average number per testis for early germ cells and separately for GSCs and goniablasts. The Wilcoxon/Mann-Whitney (U) test was used for pairwise comparison of mutant and control testes, as indicated at the bottom of the table. See also Supplementary Figure S2 for additional TUNEL assay data.

Then, we examined the effects of lacking *vasa* expression on the maintenance of testis GSCs. In wild-type males, Vasa-positive GSCs are located at the apical tip of the testis around the hub (Figure 1A). We identified the hub as a cluster of closely fitting small cells at the apical testis tip brightly expressing the marker protein Fasciclin III (FasIII) on their surface. We scored GSCs as Vasa-positive cells that directly contacted the hub and contained the α-spectrin-positive dot-like organelle, the spectrosomes, in control heterozygous testes. In the testes of *vasa* mutant males, we defined GSCs as cells in direct contact with the hub and carrying the spectrosomes (Figure 2A). We found a significant decline of GSCs in the testes of *vasa* heteroallelic mutant combinations, *vas* ^*EP812*^ */vas* ^*D1*^ and *vas* ^*EP812*^ */vas* ^*PH165*^, compared with heterozygous controls in two age groups (0 days after eclosion, *p* < 4.3E-8, and 6 days old, *p* < 2.3E-10, Wilcoxon/Mann-Whitney test, Figures 2A, B; Supplementary Figure S1B). These data indicate that Vasa is required intrinsically for the maintenance of testis GSCs.

We could expect a premature loss of GSCs according to one of two general models: a loss through programmed cell death or premature differentiation of germ cells followed by normal development of newly generated germline cysts. To estimate programmed cell death events during early spermatogenesis, we used the TUNEL assay, which detects massive DNA fragmentation associated with necrotic or apoptotic dying cells (Arama and Steller, 2006; Kibanov et al., 2013). Taking into account a rapid loss of total germline content in the testes of *vasa* mutants with aging, we performed TUNEL assay using the testes of very young males (0–1 day after eclosion) (Figure 2C; Supplementary Figure S2). We found that the average number of programmed cell death events per whole testis was 2.72 for mutants and 3.95 for heterozygous control (significant differences are found by Wilcoxon/Mann–Whitney test, *p* < 0.0012) (Figure 2D; Supplementary Figure S2). We believe that the observed difference in the average number of programmed cell death events per mutant and control testis is not biologically significant. According to our data, the total number of cases of programmed cell death was rather higher in control testes than in mutant testes. But in both cases, there are few apoptotic events per testis, and they do not have a noticeable effect on the process of spermatogenesis. Note that in the wild-type testes, apoptosis of single cysts occurs with low frequency, but it does not affect the development of adjacent cysts, and the lost cysts are rapidly replaced during the following waves of spermatogenesis (Arama and Steller, 2006; Kibanov et al., 2013). Thus, our results indicate that there is no increased frequency of total cell death events in mutant versus control testes. At the same time, programmed cell death events in GSCs and goniablasts consisted of 0.08 and 0.13 per testis in average for *vas* ^*EP812*^ */vas* ^*D1*^ mutants and heterozygous control, respectively (insignificant differences according to Wilcoxon/Mann-Whitney test, *p* < 0.356) (Figures 2C, D). We did not observe any morphological abnormalities of early germ cells, including giant nuclear and cellular sizes, in the mutant testes. Instead, we detected the presence of differentiating cysts marked with branched fusomes in the testes of *vasa* mutant males similar to the control (Figure 2C, yellow arrows). We also performed staining of the testes for the phosphorylated H2A variant (γ-H2Av), a marker for DNA double-strand breaks (DSBs). However, we reveal an accumulation of DSBs at the apical tips in the mutant testes with a frequency that is equal to that of heterozygous controls (Supplementary Figure S3), indicating that a loss of GSCs was not caused by unrepaired DNA breaks and subsequent cell death. Thus, the obtained results indicate that defects in the maintenance of GSCs in the testes of *vasa* mutant males are generally not associated with premature cell death and rather support a loss of GSCs by entering differentiation without a self-renewal.

### *Rhino* mutants phenocopy disorders of testis morphology of *vasa* mutants

Recently published data demonstrate that loss-of-function mutations in the gene encoding nuclear piRNA pathway component, *rhino (rhi),* lead to morphological and functional abnormalities in the testes of *Drosophila,* including a premature loss of GSCs, sharply decreased total germ cell content, and reduced fertility by aging (Chen et al., 2021). Thus, *rhi* mutation defects resemble those of *vasa* mutant testes. It is also known that loss-of-function mutations of another piRNA pathway component, *aubergine (aub)*, cause disturbances of GSC maintenance in the ovaries (Ma et al., 2017; Rojas-Ríos et al., 2017). Aub is autonomously required in GSCs for their self-renewal, and Aub loading by piRNAs is required for that (Rojas-Ríos et al., 2017).

**FIGURE 3:**
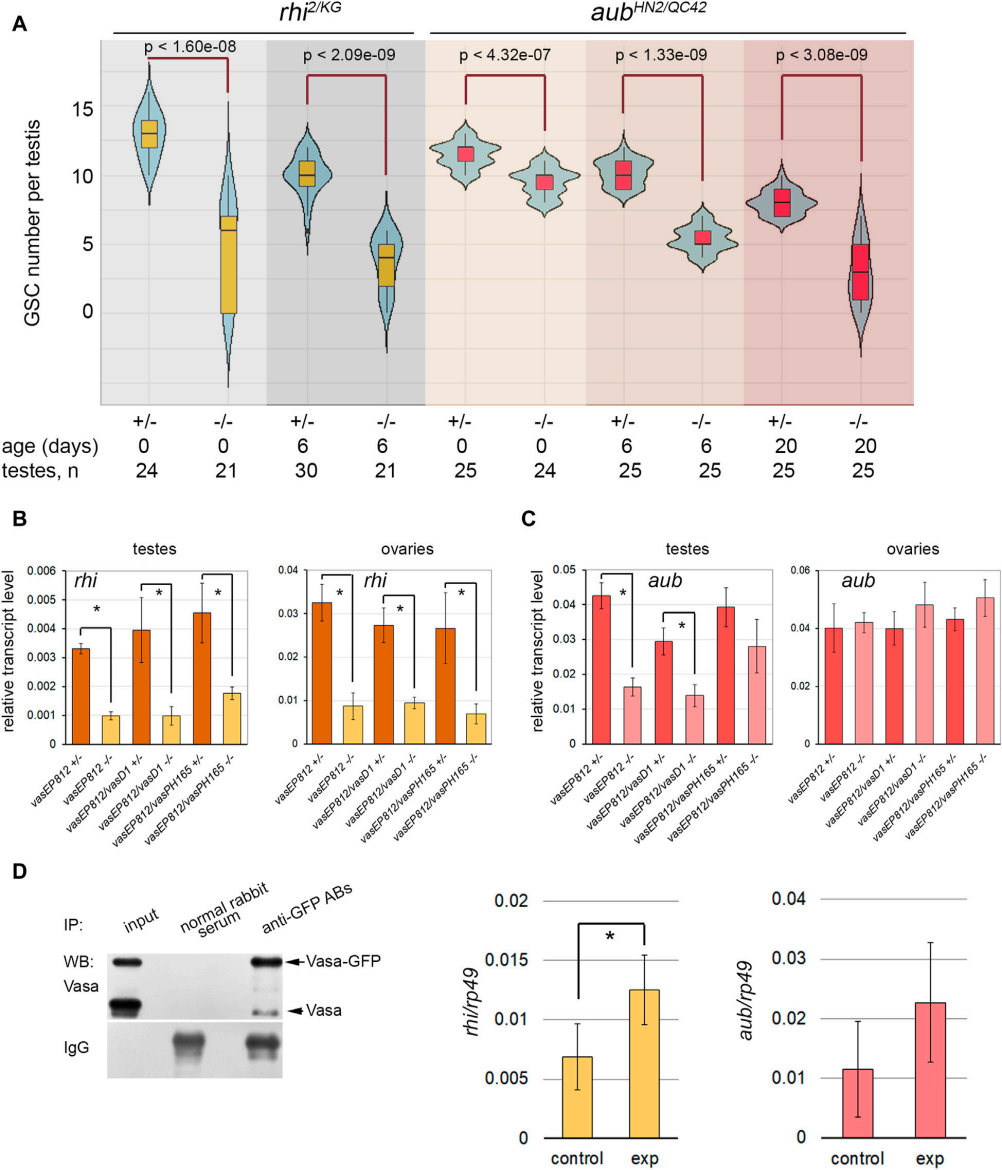
Identification of Vasa mRNA target. **(A)** Violin plots present GSC number per testis in the testes of *rhi* and *aub* mutants and heterozygous control. The medians and quartiles of GSC number sets are marked. The number of testes counted for each genotype and the age of the analyzed males are shown at the bottom. * Significant differences are found by the indicated pairwise comparison of GSC numbers in the mutant and control males of the same age (Wilcoxon/Mann-Whitney (U) test). **(B, C)** RT-qPCR analysis of transcription levels of *rhi* **(B)** and *aub* **(C)** in the testes and ovaries of *vasa* mutants. The expression levels of mRNAs were normalized to *rp49* transcripts. Error bars represent standard errors of the mean. Differences between the mutant and control heterozygous samples siblings were analyzed by Student’s t-test. The asterisks indicate significant differences between conditions; *p < 0.05. **(D)** RIP-qPCR assay for Vasa target mRNA identification. Left: Western blot analysis of immunoprecipitation of *vasa-GFP* ovarian lysate using antibodies to GFP and normal rabbit serum for control. Middle and right: RT-qPCR analysis of experimental and control RNA-immunoprecipitates with transcript-specific primers for *rhi* and *aub* with normalization to *rp49* transcripts. * The asterisks indicate that significant differences between the experiment and the control (Student’s t-test, *p* < 0.05) were found only for *rhi* transcripts.

Analyzing GSC maintenance in the testes of *rhi* ^*2/KG*^ and *aub* ^*HN2/QC42*^ males, we found that GSC number decreased with aging compared to controls in both of the cases (Figure 3A). Nevertheless, only for *rhi* mutant testes we observed defects in GSC maintenance similar to those in the *vasa* testes a week later of fly eclosion. We employed the combination of *aub* ^*HN2*^ and *aub* ^*QC42*^ mutations harboring strong and null alleles (Schüpbach and Wieschaus, 1991) to examine the function of Aub in testis GSC maintenance. However, our analysis of *aub* ^*HN2/QC42*^ testes revealed that a decline in GSC number was delayed in time compared with *vasa* or *rhi* testes, and similar effects of GSC loss were observed to the 20th day after male eclosion (Figure 3A).

To identify the relationship between Vasa and these genes, we performed RT-qPCR analysis using the gonads of *vasa* mutants. The relative level of *rhi* transcripts decreased more than three times in the testes of *vasa* mutant males (0-day-old after eclosion) compared to heterozygous control (Figure 3B). Significant differences were found for all three mutant combinations with *p* < 0.05. The level of *rhi* transcripts in the ovaries of *vasa* mutant females (3–5 days after eclosion) was also significantly reduced, *p* < 0.05 (Figure 3B). Thus, our results indicate the existence of genetic interaction between *vasa* and *rhi.* According to our data, the level of *aub* transcripts decreased in the mutant testes compared to the control, but only in the testes of *vas* ^*EP812*^ */vas* ^*EP812*^ and *vas* ^*EP812*^ */vas* ^*D1*^ males, differences were found to be significant with *p* < 0.05 (Figure 3C). The analysis of *vasa* mutant ovaries did not reveal significant differences for *aub* transcript abundance compared to control (Figure 3C). Using Western blot analysis, we defined that in the testes of 0-day-old *vasa* mutants, the level of Aub protein was sharply decreased (Supplementary Figure S4A). We observed that the level of Aub was also reduced in the ovaries of *vasa* mutant females (3–5 days after eclosion) (Supplementary Figure S4B). It should be proposed that Vasa is able to facilitate translation of *aub* mRNA or provide stabilization of Aub protein in *nuage* granules.

Since RNA helicase Vasa is known to be located only in the cytoplasmic compartment of germ cells (Snee and Macdonald, 2004; Lim and Kai, 2007; Malone et al., 2009; Kibanov et al., 2011), it cannot directly regulate gene transcription. We assumed that Vasa may specifically bind to *rhi* mRNA in gonads, promoting translation initiation and/or transcript stabilization, as previously shown for *mei-P26* mRNA in the ovaries (Liu et al., 2009). To test this hypothesis, RNA immunoprecipitation (RIP) experiments were carried out using *vasa-GFP D. melanogaster* flies expressing in germ cells the functional *vasa* copy fused with the GFP tag (Kugler et al., 2010). Since *rhi* expression in the testes is restricted to a small cohort of early germ cells from GSCs to spermatogonia (Chen et al., 2021), we performed RNA immunoprecipitation (RIP) experiments using formaldehyde cross-linked lysates of *vasa-GFP* ovaries and antibodies to GFP with a high affinity. The recovery of Vasa-GFP protein in the immunoprecipitation procedure was monitored by Western blot analysis (Figure 3D, left). RIP-qPCR analysis showed significant enrichment (*p* < 0.05) (Figure 3D, middle) of *rhi* transcripts in complex with Vasa-GFP, according to the results of three independent experiments. Thus, our data in sum suggest that RNA helicase Vasa is a positive regulator of *rhi* expression in the gonads of *D. melanogaster*.

The enrichment of *aub* mRNA in RIP-qPCR experiments turned out to be insignificant (*p* < 0.1) (Figure 3D, right). Based on previously published data, Aub colocalizes with Vasa in *nuage* granules and directly interacts with Vasa during posttranscriptional piRNA biogenesis (Kibanov et al., 2011; Webster et al., 2015; Zheng et al., 2016; Durdevic and Ephrussi, 2019). A loss of Vasa, the top hierarchical *nuage* component, causes the mislocalization of Aub in the cytoplasm in the ovaries and testes (Lim and Kai, 2007; Malone et al., 2009; Kibanov et al., 2011). Taking this into account, our results allow us to propose that Vasa contributes to the stability of Aub protein within *nuage* granules.

### Expression of transgene *rhi* copy partially suppresses premeiotic germ cell loss in the testes of *vasa* mutants

**FIGURE 4:**
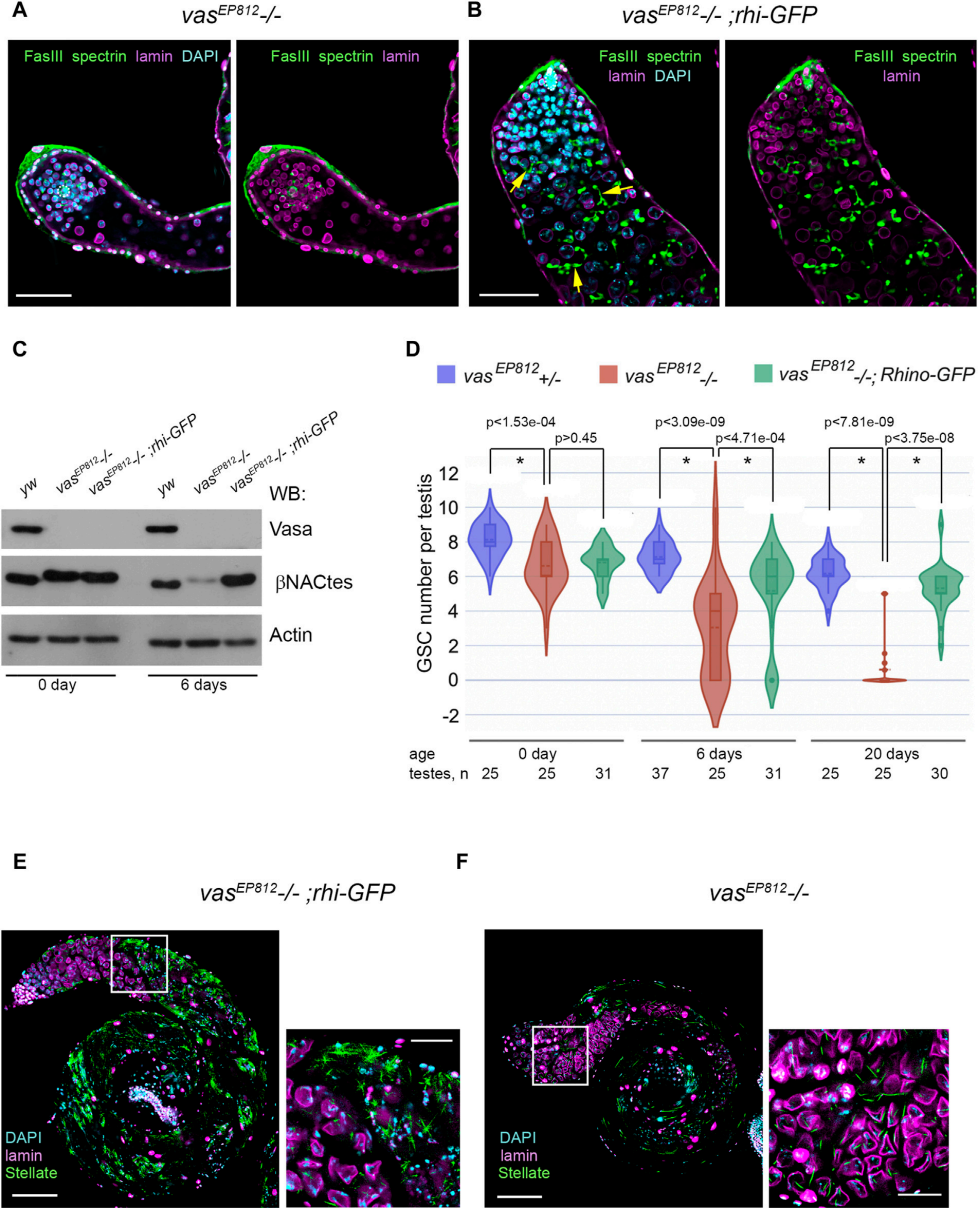
Effects of additional *rhi* expression in the testes of *vasa* mutants. **(A, B)** Immunofluorescence analysis of *vas* ^*EP812*^ *−/−* **(A)** and *vas* ^*EP812*^ *;rhi-GFP* **(B)** testes. Testes of 6-day-old males were stained with antibodies to Fasciclin III (FasIII, green), α-spectrin (green), and lamin (violet). Internal confocal slices of the immunostained whole-mount fixed testis preparations are shown. The hubs are marked by white outlines. The fusomes are indicated by yellow arrows **(B)**. Scale bars are 50 µm. **(C)** Western blot analysis of testis lysates of 0- and 6-day-old *yw*, *vas* ^*EP812*^ *−/−,* and *vas* ^*EP812*^ *;rhi-GFP* males using antibodies to Vasa, βNACtes, and Actin. Actin was used as a loading control. βNACtes is a marker of spermatocytes, which in normal circumstances constitute the most significant part of the germinal content of testes. **(D)** Violin plots present GSC number per testis in *vas* ^*EP812*^ *+/−*, *vas* ^*EP812*^ *−/−*, and *vas* ^*EP812*^ *;rhi-GFP* males. The number of testes counted for each genotype and the age of males are shown at the bottom. * Significant differences are found by indicated pairwise comparisons (Wilcoxon/Mann-Whitney (U) test). **(E, F)** Stellate expression in the *vas* ^*EP812*^ *;rhi-GFP* **(E)** and *vas* ^*EP812*^ *−/−* **(F)** testes. Testes of 0-day-old males were stained with antibodies to Stellate (green) and lamin (violet). Chromatin was stained with DAPI (blue). Images of whole testes are presented on the left. Scale bars are 100 µm. Fragments in the white boxes of the left images are present in higher magnifications on the right. Scale bars are 30 µm.

Based on our findings of genetic and molecular interactions between *rhi* and *vasa,* we investigated the potential effects of an additional *rhi* dose on spermatogenesis in the absence of *vasa* expression. We introduced the transgenic construct *rhi-GFP* (Mohn et al., 2014) in the background of the *vasa* mutation (*vas* ^*EP812*^ *−/−*) through successive crosses. We detected a high level of *rhi* transcripts in the testes of *vas* ^*EP812*^ *;rhi-GFP* males and a presence of Rhi-GFP fluorescent signal in early germ cells at the apical testis tip, corresponding to the Rhi expression pattern in the testes of wild-type males (Supplementary Figure S5). The testes of *vas* ^*EP812*^ *;rhi-GFP* males (6-day-old after eclosion) exhibited normal morphology and size and contained multiple germ cells, including spermatogonia and spermatocytes with branched fusomes. While the testes of *vas* ^*EP812*^ mutant males of the same age were reduced in size and contained a small number of germ cells (Figures 4A, B). According to Western blot analysis (Figure 4C), in the testes of 0-day-old males from the experimental and control lines, we observed an equal intensity signal of βNACtes, a marker of spermatocytes (Kogan et al., 2022), which indicates a similar filling of the testes with premeiotic germ cells. However, in the testes of 6-day-old males, βNACtes signal of similar intensity was observed only for *yw* (wild type control) and *vas* ^*EP812*^ *;rhi-GFP* males, whereas for *vas* ^*EP812*^ testes it was more than 10-fold weaker. This indicates that the amount of germ cells is sharply reduced in the testes of 6-day-old *vas* ^*EP812*^ males but remains at a level comparable to the wild type in the testes of *vas* ^*EP812*^ *;rhi-GFP* males.

To examine whether the GSC number is also restored in the testes of the *vas* ^*EP812*^ *;rhi-GFP* line, we counted them using the method described above (Figure 4D). Testes from *vas* ^*EP812*^ *−/−* males showed a rapid loss of GSCs, in accordance with data for other *vasa* mutant combinations as shown above (Figure 2B). However, for *vas* ^*EP812*^ *;rhi-GFP* males, the amount of GSCs per testis decreased more slowly and exhibited significant differences with *vas* ^*EP812*^ mutants from the 6-day age and after (Figure 4D). In sum, our data demonstrate that the expression of additional *rhi* copy ameliorates the phenotype of *vasa* mutant testes, partially restoring not only premeiotic germ cell content in the testes of *vasa* mutants but also the maintenance of GSCs. We asked whether the expression of the additional *rhi* copy in the background of *vasa* mutation affected the late stages of spermatogenesis. It is known that tandemly repeated *Stellate* genes are the main targets of the piRNA pathway in *D. melanogaster* spermatocytes (Aravin et al., 2001; Vagin et al., 2006; Kotov et al., 2019). RNA helicase Vasa is required for *Stellate* silencing, being a basic architectural and functional component of *nuage* granules where posttranscriptional *Su(Ste)* piRNA biogenesis and piRNA silencing of *Stellates* proceed (Lim and Kai, 2007; Kibanov et al., 2011). We found that in the testes of *vas* ^*EP812*^ *;rhi-GFP* males, a strong derepression of *Stellate* genes occurred with the formation of crystalline aggregates of Stellate protein (Figure 4E). It has been shown previously that the presence of more than 50–60 *Stellate* gene copies in the genome in cases of their derepression leads to complete male sterility due to strong meiotic disorders (Palumbo et al., 1994). We showed that the derepression of *Stellate* genes occurs in the testes of all three *vasa* mutant combinations; however, the expression of Stellate protein appears to be relatively low (Figure 4F; Supplementary Figure S6A), presumably owing to the premature loss of premeiotic germ cells with aging and the small number of *Stellate* copies in the genome. Compared with that in the testes of *vas* ^*EP812*^ *;rhi-GFP* males, extremely high expression of Stellate protein was observed (Figure 4E; Supplementary Figure S6B). We developed a method for the estimation of *Stellate* gene copies in the genome (see Materials and Methods for details). For *vas* ^*EP812*^ males, we determined about 19 Stellate copies; that is not enough for complete sterility (Figure 6C). We determined more than 160 *Stellate* copies for our “rescue” *vas* ^*EP812*^ *;rhi-GFP* line (Supplementary Figure S6C), obtained through several crossing rounds. We assume that both the rescue effect on germ cell maintenance and the large number of *Stellate* copies in the genome contribute to the observed testis phenotype. In accordance with these data, *vas* ^*EP812*^ *;rhi-GFP* testes demonstrated complete sterility with defects of the elongated spermatid individualization process and lacking of mature sperm in the seminal vesicles compared to *vas* ^*EP812*^ *+/−* control ones (Supplementary Figures S6D, E).

Thus, the expression of the additional *rhi* dose in the background of *vasa* mutation leads to a significant restoration of GSC number and total content of premeiotic germ cells in the testes but does not restore piRNA silencing of *Stellate* genes. This experimental model allowed us to clearly separate two distinct functions of Vasa protein in spermatogenesis of *D. melanogaster*.

### Vasa is essential for ping-pong biogenesis of piRNAs directed to *stellate* silencing

**FIGURE 5:**
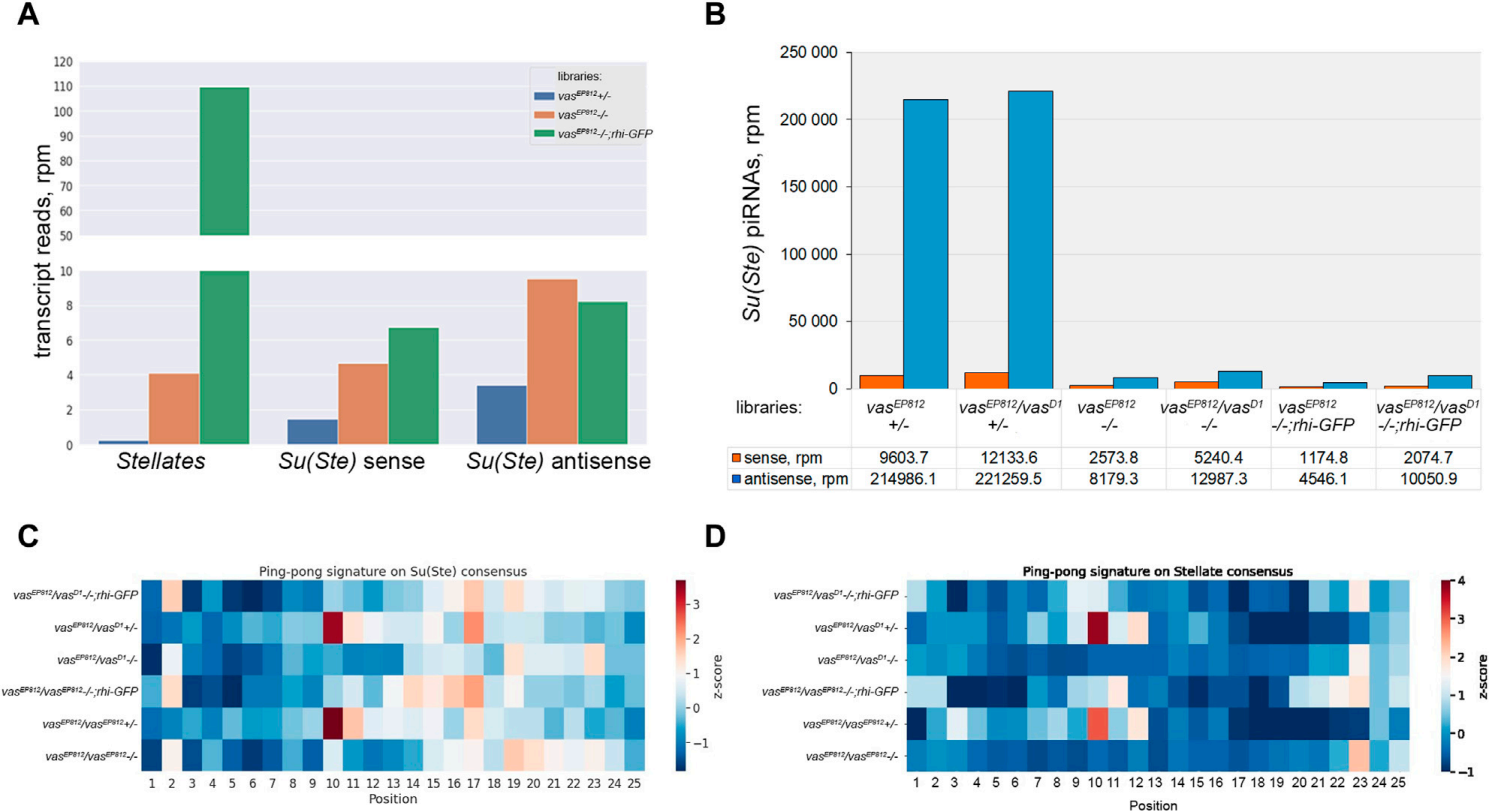
Analysis of the biogenesis of *Su(Ste)* piRNAs. **(A)** Evaluation of the expression level of *Stellate* and *Su(Ste)* repeats using RNA-seq library data. A strong increase in *Sellate* transcript abundance was found in *vas* ^*EP812*^−/− and *vas* ^*EP812*^ *;rhi-GFP* testis libraries*.* In *vasa* mutant and *vas* ^*EP812*^ *;rhi-GFP* testes, the level of antisense *Su(Ste)* transcripts was 2.8- and 2.4-fold higher than in the testes of heterozygous males. Thus, it indicates that in *vasa* mutants, the accumulation of unprocessed antisense *Su(Ste)* transcripts occurred. **(B)** Comparative analysis of the biogenesis of *Su(Ste)* piRNAs in *vasa* mutants. In the testes of *vasa* mutant males, a strong decline in antisense *Su(Ste)* piRNA production, more than 25-fold compared to heterozygous controls, was found. Antisense *Su(Ste)* piRNAs detected in the testes of *vasa* mutants presumably are produced by the phasing mechanism without the ping-pong amplification process. **(C)** Heat map presentation of the ping-pong signatures (z_10_ score) for piRNA pairs mapped on the *Su(Ste)* consensus sequence with 0–2 mm. **(D)** Heat map presentation of the ping-pong signatures (z_10_ score) for piRNA pairs mapped on the *Stellate* consensus sequence with 0–2 mm. Enrichment of ping-pong pairs mapped on *Stellate* and *Su(Ste)* sequences was found in the testes of heterozygous males but not in *vasa* mutant testes. See also Supplementary Table S1 for z score data.

To study the contribution of Vasa in *Stellate* silencing in more detail, we prepared and sequenced on the Illumina platform whole-transcriptome libraries from the testes of *vas* ^*EP812*^−/−, *vas* ^*EP812*^ *+/−,* and *vas* ^*EP812*^ *;rhi-GFP* males. To evaluate the expression level of *Stellate* and *Su(Ste)* repeats, we mapped RNA-seq library data to their consensus sequences (Figure 5A). We evidently observed a strong increase in *Sellate* transcript abundance in the testes of *vasa* mutants (up to 17.7- and 418.9-fold higher than in heterozygous control for *vas* ^*EP812*^−/− and *vas* ^*EP812*^ *;rhi-GFP* males, respectively). We found that in *vasa* mutant and *vas* ^*EP812*^ *;rhi-GFP* males, the level of antisense *Su(Ste)* transcripts was 2.8- and 2.4-fold higher than in the steady-state conditions in heterozygous males (Figure 5A). Thus, in the background of *vasa* mutation, the accumulation of unprocessed *Su(Ste)* transcripts occurred due to failures of their processing into mature piRNAs. At the same time, the additional *rhi* dose in the testes of *vas* ^*EP812*^ *;rhi-GFP* males did not significantly affect the level of *Su(Ste)* transcripts (Figure 5A), according to previously published data (Chen et al., 2021).

It is known that *Stellate* genes are transcriptionally silent in early germ cells, and they start to express in spermatocytes (Aravin et al., 2004; Egorova et al., 2009; Venkei et al., 2023; Shao et al., 2024). However, antisense transcription of *Su(Ste)* repeats was observed at an earlier stage in GSCs and spermatogonia (Aravin et al., 2004; Venkei et al., 2023; Shao et al., 2024). According to a recent study, antisense *Su(Ste)* transcripts undergo processing into piRNAs in early germ cells via Vasa-independent phased piRNA biogenesis triggered by maternally inherited transposon *hoppel*-derived piRNAs (Venkei et al., 2023). To analyze a generation pattern of *Su(Ste)* piRNAs, we prepared and sequenced libraries of 18–29 nt small RNAs isolated from the testes of *vasa* mutants, control heterozygous males, and males with the transgenic construct *rhi-GFP* in the background of *vasa* mutations.

We selected piRNA fractions (23–29 nt) of small RNA libraries and mapped them on the *Su(Ste)* consensus. *Su(Ste)*-mapped reads (with 0–2 mismatches) are present in the control heterozygous testis libraries in large amounts (Figure 5B). The ratios of antisense and sense *Su(Ste)* piRNAs were found in the range 18.2–22.4 (Figure 5B), which is in accordance with our data published earlier (Kotov et al., 2019). In the testes of *vasa* mutant males, we found a strong decline of antisense *Su(Ste)* piRNA production more than 25-fold compared to controls (Figure 5B). Similar results were obtained by analysis of the libraries from the testes of *vasa* mutants carrying *rhi-GFP* transgene, where antisense *Su(Ste)* piRNA amounts were found to be decreased from 22- to 47.3-fold relative to heterozygous controls (Figure 5B). Thus, antisense *Su(Ste)* piRNAs detected in the testes of *vasa* mutants appear to be those produced by the phasing mechanism. Despite presence, their amount appears to be insufficient to repress *Stellate* genes. The ping-pong amplification process is known as an adaptive mechanism of the piRNA biogenesis in the germline, ensuring effective repression of targets (Brennecke et al., 2007; Gunawardane et al., 2007). Accordingly, our analysis revealed ping-pong signatures (the prevalence of 10 nt overlapping sense and antisense piRNA pairs) for piRNAs mapped on both *Su(Ste)* and *Stellate* in the testes of control heterozygous males *vas* ^*EP812*^ *+/−*, *vas* ^*EP812*^ */vas* ^*D1*^ *+/−*, with z_10_ score values are 3.41 and 3.69 for *Su(Ste*)*,* 3.77 and 3.04 for *Stellate,* respectively (Figures 5C, D; Supplementary Table S1). In the case of *vasa* mutants, including those with transgene *rhi-GFP* copy, generation of piRNAs *via* the ping-pong process does not occur (Figures 5C, D).

Taken together, our data suggest that the presence of maternally inherited transposon-derived piRNAs for the initiation of *Su(Ste)* piRNA generation by the phasing process itself is not enough for perfect repression of *Stellates*. Vasa-dependent amplification ping-pong process is strongly required for effective *Su(Ste)* piRNA biogenesis and *Stellate* silencing.

## Discussion

A large body of evidence indicates that Vasa plays an essential role in *Drosophila* oogenesis during the early and late stages (Lasko and Ashburner, 1990; Czech et al., 2013; Durdevic et al., 2018; Durdevic and Ephrussi, 2019). Females with *vasa* null alleles lay a few embryos; they do not form germ cells, and adult offspring possess abdominal defects and defects of gonad development (Lasko and Ashburner, 1988; Hay et al., 1988). The functions of Vasa in early oogenesis are also associated with the maintenance and self-renewal divisions of GSCs (Dehghani and Lasko, 2015). At the same time, Vasa functions in fly spermatogenesis have remained unclear to date. Moreover, it is supposed that Vasa is not required for spermatogenesis since young males, mutants for *vasa,* have been found to be fertile (Lasko and Ashburner, 1988; Dehghani and Lasko, 2017). This circumstance for a long time has supported the hypothesis about sexual dimorphism of Vasa functions in *D. melanogaster*, similar to that showing for mouse Vasa homologue MVH in *Mus musculus* (Tanaka et al., 2000; Kuramochi-Miyagawa et al., 2010).

Here we first demonstrated that RNA helicase Vasa is an essential protein for GSC maintenance in *Drosophila* testes. In loss-of-function *vasa* mutant males, the number of GSCs was significantly reduced in comparison with heterozygous control males of the same age (Figure 2B). The premature loss of GSCs caused a reduction in the total population of differentiating germ cells, suppression of spermatogenesis, and a sharp fertility decline with aging (Figures 1C–E). The loss of GSCs was not associated with increased incidences of programmed cell death (Figures 2C, D) and was rather a consequence of their poor maintenance in the niche. The fertility of young *vasa* males in a short time after eclosion indicates that age-dependent loss of GSCs appears to be a cell-autonomous process at the level of a single GSC, which loses contact with the niche and enters differentiation. In oogenesis, Vasa is known as a translational activator of specific transcripts, such as *nanos, gurken*, and *mei-P26* mRNAs (Gavis et al., 1996; Styhler et al., 1998; Tomancak et al., 1998; Johnstone and Lasko, 2004; Liu et al., 2009). Other protein components of the piRNA pathway, Aub and Piwi in *Drosophila* and MIWI and MILI in mice, have been shown to involve translational regulation of protein-coding genes. Aub and MIWI physically interact with the components of translation machinery such as the cap-binding complex through their interactions with subunits of the eIF3 complex, eIF4E, PABP, and Aub is also able to recruit poly(A) polymerase Wispy for the stabilization of specific transcripts in the embryonic germ plasm (Unhavaithaya et al., 2009; Dufourt et al., 2017; Dai et al., 2019; Ramat et al., 2020; Ramat and Simonelig, 2021). Vasa presumably is a component of a protein-protein interaction network, including Aub, Rm62, PABP, and eIF3 proteins, that have been shown to control germ cell development and maintenance (Ma et al., 2017; Ramat et al., 2020). A set of factors of translation initiation involved in the formation of the pre-initiation complex, eIF2, eIF3, and the eIF4E-4G cap-binding complex has been shown to be coimmunoprecipitated with Vasa from *Drosophila* ovaries (Durdevic and Ephrussi, 2019; Bansal et al., 2020). However, the exact mechanism and direct mRNA targets of translational regulation with the aid of Vasa remain obscure to date, and nothing is known about Vasa targets in *Drosophila* spermatogenesis.

Here we uncovered a direct relationship between Vasa and *rhi* (Figures 3B, D). Significantly decreasing *rhi* transcript levels in *vasa* mutant testes and the association of Vasa with *rhi* transcripts indicate its involvement in the positive regulation of *rhi* expression at the posttranscriptional level. Rhi is an important piRNA pathway protein, a key component of the RDC complex, responsible for non-canonical transcription from double-stranded piRNA clusters (Klattenhoff et al., 2009; Le Thomas et al., 2014; Mohn et al., 2014; Zhang et al., 2014). Recently published data demonstrate that mutations *rhi* exhibit morphological and functional abnormalities in the testes (Chen et al., 2021) that resemble those observed in *vasa* mutant testes in our study, including premature loss of GSCs and rapid decline of male fertility with age. Taking into account the effect of Vasa on *rhi* expression, we generated flies carrying the construct with the additional copy of *rhi* in the absence of *vasa* expression. This results in the restoration of normal testes morphology and the maintenance of GSCs for a long time, but not the restoration of male fertility (Figure 4; Supplementary Figure S6E).

The experiment for “rescuing” testis morphology with the aid of an additional *rhi* dose allowed us to identify two most critical developmental points of necessity in Vasa function for spermatogenesis. First is the maintenance of GSCs associated with positive regulation of *rhi* expression. It remains unclear how Rhi functions in maintaining GSCs in the testes. We assume that this function is not directly related to piRNA repression of TEs. In the recent study, it has been shown that, apart from piRNA clusters, Rhi protein is recruited to hundreds of chromatin sites in the ovaries with the aid of ovary-specific partner protein Kipferl (Baumgartner et al., 2022). The genetic and functional regulatory relationship established between Vasa and Rhi, components of the piRNA system, supports the assumption that the piRNA pathway could be involved in gene regulatory process directed to GSC maintenance in the testes of *D. melanogaster*. However, further studies are needed to explore the molecular mechanism of this process.

The second critical point for ensuring male fertility in *D. melanogaster* is Vasa-dependent *Stellate* silencing in premeiotic spermatocytes. According to the current view, antisense transcription of Y-linked *Su(Ste)* repeats begins in early germ cells of testes (Aravin et al., 2004; Venkei et al., 2023; Shao et al., 2024). Note that antisense transcription from the *Su(Ste)* cluster does not require Rhi, as has been shown previously (Chen et al., 2021). *Su(Ste)* is not a Rhi-dependent dual-stranded piRNA cluster with a non-canonical transcription mode described in *Drosophila* ovaries (Le Thomas et al., 2014; Mohn et al., 2014; Zhang et al., 2014). Transcription of sense *Su(Ste)* RNAs is carried out from the classical promoter region in a later developmental period than antisense RNAs (Aravin et al., 2004; Olenkina et al., 2012; Shao et al., 2024). Phasing-based production of antisense *Su(Ste)* piRNAs in early germ cells appears to initiate with the aid of maternally inherited piRNAs derived from transposon *hoppel* inserted in the promotor region of *Su(Ste)* repeats (Venkei et al., 2023). The authors of this paper emphasize that abundant antisense *Su(Ste)* piRNAs are produced by the phasing before starting transcription of their target *Stellate* genes. However, other sources demonstrate a significant overlap between the transcription windows of *Su(Ste)* and *Stellate* rather than their spatiotemporal separation (Shao et al., 2024). Taking advantage of small RNA library data analysis, we revealed that only a small amount of antisense *Su(Ste)* piRNAs were accumulated in *vasa* mutant testes, with a more than 25-fold reduction compared to heterozygous control (Figure 5B). Indeed, these piRNAs have no characteristic ping-pong signatures, indicating in favor of their processing by the phasing mechanism (Figure 5C). Because *Stellate* derepression is clearly observed in *vasa* mutant testes (Figure 4F; Supplementary Figure S6A), the amount of these *Su(Ste)* piRNAs is not enough for *Stellate* silencing. Note that in the testes of males carrying the additional copy of *rhi* in the absence of *vasa* expression, we revealed the similar disruption of antisense *Su(Ste)* piRNA biogenesis and the absence of the ping-pong process (Figures 5B–D). Thus, using comparative analysis of small RNA libraries, we confirmed that Vasa-dependent processing of abundant *Su(Ste)* piRNAs via the ping-pong mechanism is required for *Stellate* silencing (Figure 5C; Supplementary Table S1). This processing is Rhi-independent in accordance with previous findings (Chen et al., 2021).

Here we found a strong correlation between *vasa* deficiencies in the testes and ovaries and the decreased level of Aub protein, but not its transcripts (Figure 3C; Supplementary Figure S4). As indicated above in the testes of *vasa* mutant males, a strong, more than 25-fold reduction of antisense *Su(Ste)* piRNA production compared to heterozygous controls occurs (Figure 5B). A loss of Vasa, the top hierarchical *nuage* component, causes the mislocalization of Aub and AGO3 in the cytoplasm in the ovaries and testes (Lim and Kai, 2007; Malone et al., 2009; Kibanov et al., 2011; Kotov et al., 2014). Impaired *nuage* granule formation leads to a decline of posttranscriptional piRNA biogenesis (Lim and Kai, 2007; Kibanov et al., 2011). It is also shown that unloaded Aub with defective PAZ domain is unable to bind mRNA targets and did not localize to the germ plasm (Barckmann et al., 2015). We assume that empty Aub that is distributed in the cytoplasm undergoes a premature elimination. Note that several studies reported that unloaded ARGONAUTE family proteins undergo selective degradation in various species (Derrien et al., 2012; Martinez and Gregory, 2013; Kobayashi et al., 2019a; b). E3 ubiquitin-protein ligase Iruka recognizes miRNA-empty AGO1 in *Drosophila* and triggers its degradation via polyubiquitination of lysine residues across practically all domains, especially K514 in the linker two domain (Kobayashi et al., 2019a). Elimination of dysfunctional AGO1 that is not able to bind miRNA by proteasomal or autophagic mechanisms ensures a quality control of miRNA-mediated silencing (Kobayashi et al., 2019a; b). For unloaded PIWI subfamily of ARGONAUTE proteins, such mechanisms of elimination have not been shown yet, and this needs further studies. However, murine PIWI (MIWI) in late spermatids is ubiquitinated and degraded through the proteasome system, indicating a feedforward mechanism for coordinated elimination of the MIWI/piRNA machinery at the specific developmental stage (Zhao et al., 2013). Taking that into account, we propose that Vasa contributes to the stability of Aub protein by recruiting it into the *nuage* granules for assembling functional piRNA-Aub complexes and providing piRNA silencing of mRNA targets. Here we showed that RNA helicase Vasa essentially contributes to male fertility in *D. melanogaster* at least on two distinct stages: GSC maintenance in early spermatogenesis and piRNA-dependent *Stellate* silencing need for correct passage through meiosis in spermatocytes. Our study uncovers a novel mechanistic link involving Vasa and Rhi in a regulatory network that mediates GSC maintenance. The open questions include whether observed relationships indicate a possible role of piRNA silencing in this important developmental process.

## Materials and methods

### Fly stocks and genetics

Germinal tissues of adult *D. melanogaster* males and females generally raised at 23°C were used. We used following *Drosophila* stocks: *vas* ^*EP812*^ *(vas* ^*LYG2*^) mutant strain is originated as a result of P-element insertion into the last *vig (vasa intronic gene)* exon, adjacent to the 3′-splice site (Ashburner et al., 1999); *vas* ^*D1*^ (*b* ^*1*^ *vas* ^*3*^ */CyO*) loss-of-function allele was obtained using ethyl methanesulfonate mutagen with no changes in coding *vasa* sequence (Lasko and Ashburner, 1990); *vas* ^*PH165*^ allele was obtained by imprecise excision of P-element, resulting in a 7343bp deletion removing the entire *vasa* coding region (Styhler et al., 1998). Flies with heteroallelic mutation combinations *vas* ^*EP812*^ */vas* ^*PH165*^, and *vas* ^*EP812*^ */vas* ^*D1*^ as well as homozygotes *vas* ^*EP812*^ males were chosen for our experiments. *yw* flies *(Df(1)w* ^*67c23(2)*^ *y*)*,* was used as wild-type controls. For RIP-PCR experiments we used *yw* strain carrying transgene construct *vas-GFP* (*vas* ^*cKa.GFP*^) under the control of endogenous *vasa* promoter kindly provided by P. Lasko (Kugler et al., 2010). The following fly stocks were also used: *aub* ^*QC42*^ (BDSC4968), *aub* ^*HN2*^ (BDSC8517), were obtained from the Bloomington *Drosophila* Stock Center (Indiana University, IN, USA); *rhi* ^*2*^ *(rhi* ^*02086*^) and *rhi* ^*KG*^ *(rhi* ^*KG00910*^) lines were kindly provided from W. Theurkauf Lab. *Rhi-GFP* line (VDRC313340) were obtained from Vienna *Drosophila* Resource Center (Vienna, Austria); GFP-tagged *rhi* is a previously described transgene constructed by inserting N-terminal GFP into genomic BACs by recombineering (Mohn et al., 2014). *cry* ^*1*^ line, *ywf/c(1)DX, yf/cry* ^*1*^ *B* ^*S*^ *, Yy* ^*+*^, carries a deletion of the bulk of the *Su(Ste)* locus on the Y chromosome.

### Immunofluorescence staining, TUNEL assay and confocal microscopy

Testes of adult males were dissected in phosphate-buffered saline (PBS) at 4°C, washed with PBT (1×PBS, 0.1% Tween 20) and fixed in 3.7% formaldehyde and PBT for 30 min at room temperature. All the following procedures were carried out as described previously (Kibanov et al., 2013). Staining was detected by laser scanning confocal microscopy using a Zeiss Axio Observer LSM 900 machine (Carl Zeiss). All images were taken with a z-resolution of 1 µm. The obtained pictures were imported into Imaris 5.0.1 (Bitplane AG) for subsequent processing. Counting of spectrosomes and the number of testis GSCs on the confocal images was carried out using Imaris software. The Click-iT TUNEL Alexa Fluor imaging assay kit (Invitrogen) was used for detection of DNA breaks that associated with necrotic or apoptotic cell death according to the published protocol (Kibanov et al., 2013). TUNEL signal of whole cyst of germ cells was estimated as a single event. *p*-values for pairwise comparison in control and experimental testes were calculated using Wilcoxon/Mann–Whitney (U) test.

### Antibodies

The following antibodies were used for immunofluorescence staining: a mix of murine monoclonal anti-Lamin Dm0 ADL67.10 and ADL84.12 antibodies (Developmental Studies Hybridoma Bank, University of Iowa (DSHB)), 1:500; rabbit polyclonal anti-Lamin antibodies (Osouda et al., 2005), 1:500; rat monoclonal anti-Vasa antibody (DSHB), 1:100; murine monoclonal anti-α-spectrin 3A9 antibody (DSHB), 1:200; murine monoclonal anti-Fasciclin III 7G10 antibody (DSHB), 1:25; rabbit polyclonal anti-γH2Av pS137 antibodies (Rockland), 1:100. Alexa Fluor-labeled secondary goat anti-rat IgG, goat anti-rabbit IgG, and goat anti-mouse IgG (Invitrogen) were used as secondary reagents at a dilution of 1:500. DAPI (4′,6-diamidino-2-phenylindole) (Sigma) was used for chromatin staining.

For RNA immunoprecipitation (RIP) experiments, rabbit polyclonal anti-GFP antibody ab6556 (Abcam) was used.

For Western blot analysis, the following antibodies were used: murine monoclonal anti-β-Actin antibody ab8224 (Abcam), 1:2000; rat monoclonal anti-Vasa antibody (DSHB), 1:2000; rabbit polyclonal anti-VIG 1803 antibodies (Gracheva et al., 2009), 1:500; murine monoclonal anti-Aub 4D10 antibody (Nishida et al., 2007), 1:1500; rabbit polyclonal βNACtes antibodies (Kogan et al., 2022), 1:1000; murine polyclonal anti-Stellate antibodies, 1:500, (Egorova et al., 2009). Samples were resolved by SDS-PAGE and blotted onto PVDF membrane Immobilon-P (Sigma). Alkaline phosphatase-conjugated anti-mouse, anti-rabbit, anti-rat and anti-goat antibodies (Sigma) were used as secondary reagents at a dilution of 1:20,000. Blots were developed using the Immun-Star AP detection system (Bio-Rad Laboratories). All experiments were performed at least in triplicate with independent preparations of testis or ovarian lysates.

### RNA extraction, reverse transcription and quantitative PCR

Total RNA was isolated from sets of 50–100 pairs of dissected testes or 30–50 pairs of ovaries, using TRIzol Reagent (Invitrogen) according to the manufacturer’s recommendations. cDNA was synthesized using random hexamers and SuperScript II reverse transcriptase (Invitrogen). cDNA samples were analyzed by real-time quantitative PCR using the incorporation of SYTO-13 (Invitrogen). Thermal cycling consisted of 5 min at 95°C, followed by 45 cycles of denaturation (94°C, 20 s), annealing (64°C, 20 s), extension (72°C, 20 s), and a final extension of 5 min at 72°C. We used *rp49 (rpL32)* as a loading control. All experiments were performed with at least three independent RNA samples; each sample was analyzed in duplicate. The following primers were used for RT-qPCR and RIP-RT-qPCR: *rp49* fw 5′-ATG​ACC​ATC​CGC​CCA​GCA​TAC-3′, rev 5′-GCT​TAG​CAT​ATC​GAT​CCG​ACT​GG-3′; *aub* fw 5′-CAT​GAG​TGA​ACA​TAC​CAG​GCT​GAA-3′, rew 5′-GCG​GAG​TCC​AGC​TCG​ATG​TT-3′; *rhi* fw 5′-CGG​TTT​TCC​GAA​CGA​GAA​C-3′, rew 5′-CGG​CCT​TCC​GAT​GCA-3′.

### Copy number of *Stellate* genes estimation

The number of X-linked *Stellate* genes in the genome was assessed by quantitative PCR with genomic DNA using highly specific *Stellate* primers, the efficiency of which was previously tested and compared with the efficiency of *rp49* primers (normalization control). In these experiments, genomic DNA of both males and females of *D. melanogaster* was used as an additional control; the obtained value of the relative number of *Stellate* genes for males was 2-fold lower compared to females. *Stellate* primers: fw 5′-GAT​TGG​TTC​CTC​GGG​ATC​AA-3′, rev 5′-CCG​TAC​AAC​AAG​CCA​GAG​GAA​CT-3′.

### Fertility tests

Sets of 25–30 experimental males and their heterozygous siblings as a control were analyzed for their fertility. Individual adult male (0 days after eclosion) was placed with three virgin *yw* females 4- to 6-day-old for 5 days in separate vials at 25°C. After that the parent flies were removed from the vial. The males were translocated in other vials with new three virgin females for the next 5 days up to six times. The adult progeny in each vial was counted within 13–18-day interval after mating. The tests were carried out at 25°C. The flies were subjected to a light–dark cycle of 12:12 h. At the end of the experiments, the offspring from one male were counted per female for each time period.

### RNA immunoprecipitation

Ovaries of *vas-GFP* flies 3–5 days after eclosion were dissected in ice-cold phosphate-buffered saline (PBS). Freshly dissected gonads were cross-linked in PBS supplemented with 0.25% formaldehyde for 30 min, were washed by three times for 5 min and were stored at −70°C until further use. Total cellular lysates were obtained from 500 pair of cross-linked ovaries using pre-chilled Dounce homogenizer on ice in a cold lysis buffer (50 mM Tris-HCl pH 8.0; 100 mM KCl; 2 mM MgCl_2_; 1 mM DTT; 0.5% Nonidet P-40 (NP-40); in the presence of 1% protease and phosphatase inhibitors (Sigma) with the addition of 2% Ribolock (Thermo Fisher Scientific) by two series of 100 strokes of pestle A with a 5 min incubation between the series. The lysates were transferred to 1.7-mL polypropylene microcentrifuge tubes and were precipitated at 5000 *g* for 10 min at 4°C. The supernatant fractions (crude extracts) were cleared by subsequent centrifugation at 16,000 *g* for 20 min at 4°C. The clarified lysates were transferred to new tubes and diluted to protein concentration of 7–10 mg/mL with lysis buffer. We used 50 µL of 50%-slurry of Protein A beads (Invitrogen) for each tube for immunoprecipitation. The beads were previously washed two times with 500 µL cold PBS supplied by Tween 20 (1 × PBS; 0.1% Tween 20 (PBST)) and incubated with anti GFP-antibodies (Abcam) or normal rabbit serum for negative control in 200 µL PBST 20 min at room temperature on the rotator. The antibody-coated beads were washed and pre-equilibrated with lysis buffer. Then the equal volumes of clarified lysates were added to the experimental and control tubes for immunoprecipitation. After incubation 40 min at RT on the rotator, washing was performed three times with 1 mL washing buffer NT2 (50 mM Tris-HCl, pH 8.0; 150 mM NaCl; 0.5 mM DTT; 0.3% NP-40), three times with 1 mL washing buffer NT3-Urea (50 mM Tris-HCl, pH 8.0; 150 mM NaCl; 1M urea; 0.5 mM DTT; 0.3% NP-40) and one time NT2 buffer. Small portions of immunoprecipitated material from each tube were put aside for Western blot analysis. For that the bound material was eluted from the beads by boiling in 30 μL 2×sample buffer containing 0.2 M DTT. Samples were subsequently resolved by % SDS-PAGE and blotted onto PVDF membrane Immobilon-P (Sigma). The rest parts of the material were subjected to proteolysis to remove a bulk of proteins performed *on-bead* in solution of 4 mg/ml Proteinase K (Promega) in PK2 buffer (10 mM Tris-HCl pH 7.5; 50 mM NaCl; 5 mM EDTA; 0.5% SDS; 1 mM DTT; in the presence of 2% Ribolock) for 2 hours at 55°C with extensive agitation. After that the tubes were subjected by incubation for 10 min at 65°C for removing of cross-linking between RNA and peptide material. Soluble fractions were separated from beads and were processed with TRIzol LS reagent (Thermo Fisher Scientific) followed by RNA isolation. RT-qPCR analysis was performed as mentioned above.

### Library preparation

Total RNA for RNA-seq libraries were isolated from *vas* ^*EP812*^ *−/−*, *vas* ^*EP812*^ *+/−*, and *vas* ^*EP812*^ *−/−; rhi-GFP D. melanogaster* gonads with ExtractRNA (Evrogen). rRNA was depleted with Dynabeads MyOne Streptavidin C1 (Thermo Scientific Fisher) conjugated with synthesized anti-rDNA oligos. RNA-seq libraries were generated using the NEBNext Ultra™ II Directional RNA Library Prep Kit for Illumina (#E7760, NEB) according to the manufacturer’s instructions. Rhibo-depleted RNA-seq libraries were sequenced in 100 bp single-end mode on NovaSeq 6000 platform. Small RNAs of 18–29 nt in size from gonads of *D. melanogaster vas* ^*EP812*^ *−/−, vas* ^*EP812*^ *+/−, vas* ^*EP812*^ *−/−; rhi-GFP*, *vas* ^*EP812*^ */vas* ^*D1*^, mix of *vas* ^*EP812*^ */+* with *vas* ^*D1*^ */+*, and *vas* ^*EP812*^ */vas* ^*D1*^ *; rhi-GFP* were isolated with TRIzol Reagent (Invitrogen). Small RNA fraction (19–29 nt) was purified by 15% polyacrylamide gel and libraries were prepared using NEBNext Multiplex Small RNA Sample Prep Set for Illumina (E7300S) and sequenced on NovaSeq 6000 in 50 bp single-end mode.

### Bioinformatics analysis

Quality control of the sequenced libraries was carried out using the FastQC tool. For subsequent analysis, only reads with fastq score >30 were used. To evaluate the expression level of the *Stellate* and *Su(Ste)* loci RNA-seq libraries were mapped to consensus sequences using bowtie version 1.1.0 (-l 150). Mapped reads were counted and normalized to the library depth, rpm. To evaluate the expression level of *rhi* RNA-seq libraries were pseudo-aligned to *D. melanogaster* transcriptome (GCF_000001215.4) by kallisto (v.0.50.0) with following parameters--single -l 100 -s 0.1. Quantified abundances of transcripts (transcript per million, tpm) for protein-coding genes were imported with tximport (v.1.24.0). Adapter sequences from small RNA libraries were removed by cutadapt 2.8. After quality control with FastQC, rRNA, snRNA, snoRNA, microRNA and tRNA were filtered from the libraries and piRNA were selected by size (23–30 nt). To evaluate the piRNA level and analyze ping-pong signature of piRNAs for *Stellate* and *Su(Ste)* filtered piRNA fraction were mapped to consensus sequences using bowtie with o-2 mm (-l 30). Mapped reads were normalized to the microRNA level. Ping-pong signatures were calculated using a signature. py script (Antoniewski, 2014).

## Data Availability

The datasets presented in this study can be found in online repositories. The names of the repository/repositories and accession number(s) can be found below: https://www.ncbi.nlm.nih.gov/geo/, GSE269987 and GSE269988.
