# Supplementary material for: Essential functions of RNA helicase Vasa in maintaining germline stem cells and piRNA-guided Stellate silencing in Drosophila spermatogenesis
Source: Front Cell Dev Biol. 2024 Aug 9;12:1450227. doi: 10.3389/fcell.2024.1450227 (PMC11341464; doi:10.3389/fcell.2024.1450227)
Supplement: Supplementary file 1 [file DataSheet1.PDF]

Supplementary material

for the paper

Essential functions of RNA helicase Vasa in maintaining germline stem cells and piRNA-guided *Stellate* silencing in *Drosophila* spermatogenesis

Vladimir E. Adashev[1†], Alexei A. Kotov[1†], Sergei S. Bazylev[1], Ilia A. Kombarov[1], Oxana M. Olenkina[2], Aleksei S. Shatskikh[1], and Ludmila V. Olenina[1]

[1]*Laboratory of Functional Genomics, Koltzov Institute of Developmental Biology, Russian Academy of Sciences, 119334 Moscow, Russia;*

[2]*Department of Molecular Mechanisms for Realization of Genetic Information, National Research Centre "Kurchatov Institute", Moscow 123182, Russia;*

[†] *These authors contributed equally to this work and share first authorship*

***** *correspondence: olenina_ludmila@mail.ru; Tel: +7 499 1960809; (L.V.O.)*

File contains Figures S1-S6 with Figure legends.

Figure S1

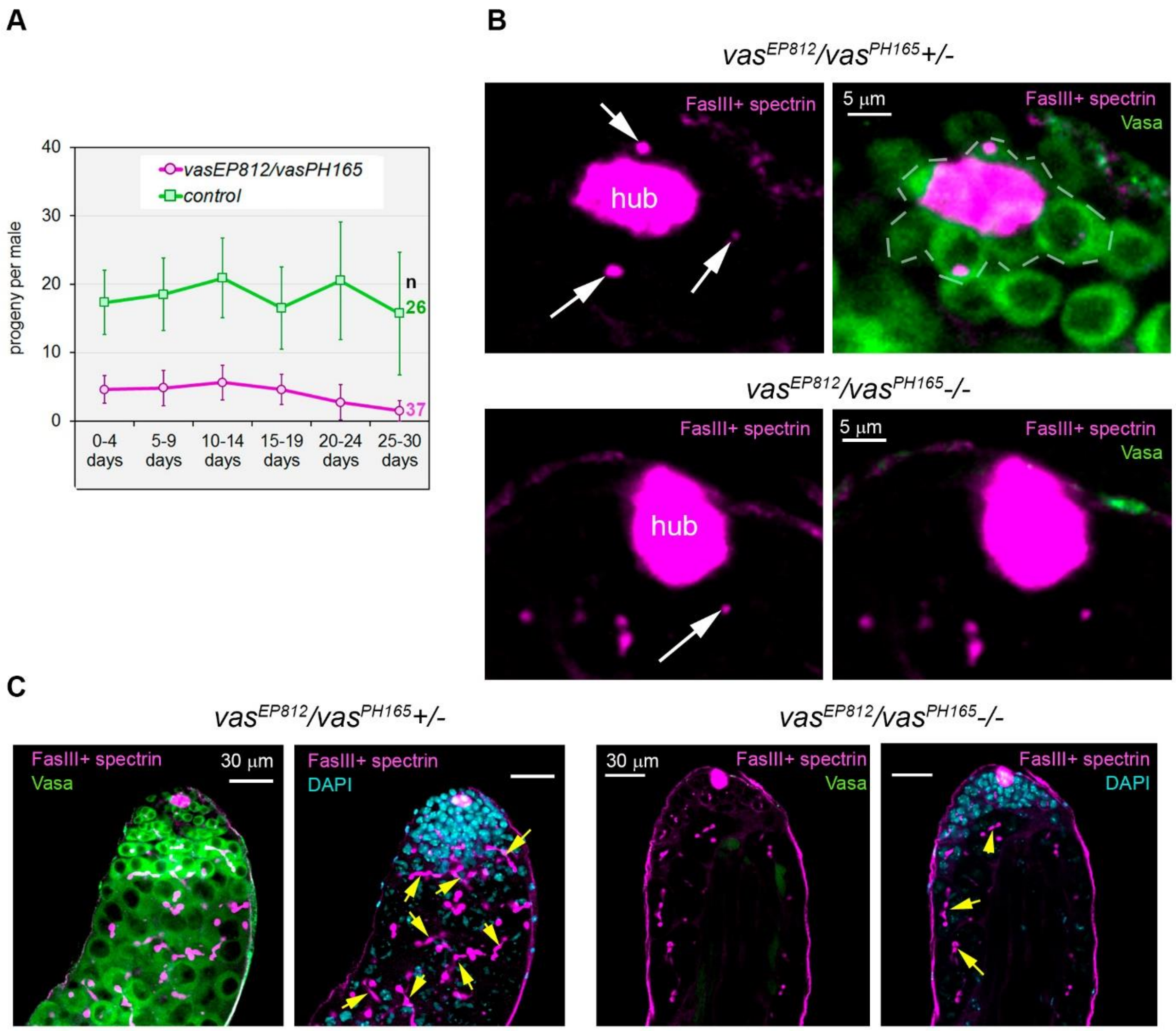

Figure S1. (**A**) Fertility test of *vasa* mutant males with *vas*^{EP812}/*vas*^{PH165} heteroallelic combinations (violet lines) in comparison with their heterozygous siblings (green lines). The average adult offspring number of male per day with standard errors is presented for indicated time intervals after parent male eclosion. The number of examined males is indicated. See Materials and Methods for details. (**B**) GSCs in the testes of *vasa* mutants and control heterozygous siblings. Testes of mutant *vas*^{EP812}/*vas*^{PH165} and heterozygous control males were immunostained with Vasa (green), Fasciclin III (violet, marker of hub), α-spectrin (violet) antibodies, and DAPI (blue) staining. White arrows indicate spectrosomes, dot-like GSC-specific organelles located near the hub. Single confocal slices of testis tip images are shown. Vasa-stained GSCs around the hub are outlined for testis of heterozygous control. (**C**) A loss of germ cells in the testes of *vas*^{EP812}/*vas*^{PH165} heteroallelic mutants. Left: testes of heterozygous control males exhibited a wild-type phenotype. Right: testes of *vas*^{EP812}/*vas*^{PH165} heteroallelic mutants partially lost germline content, as indicated by a decrease in fusome number (yellow arrows). Whole-mount fixed testes were immunostained with Vasa (green) and Fasciclin III (violet) and α-spectrin (violet) antibodies, and chromatin was stained with DAPI (blue). Internal confocal slices with the apical parts of the testes oriented upward are shown.

Figure S2

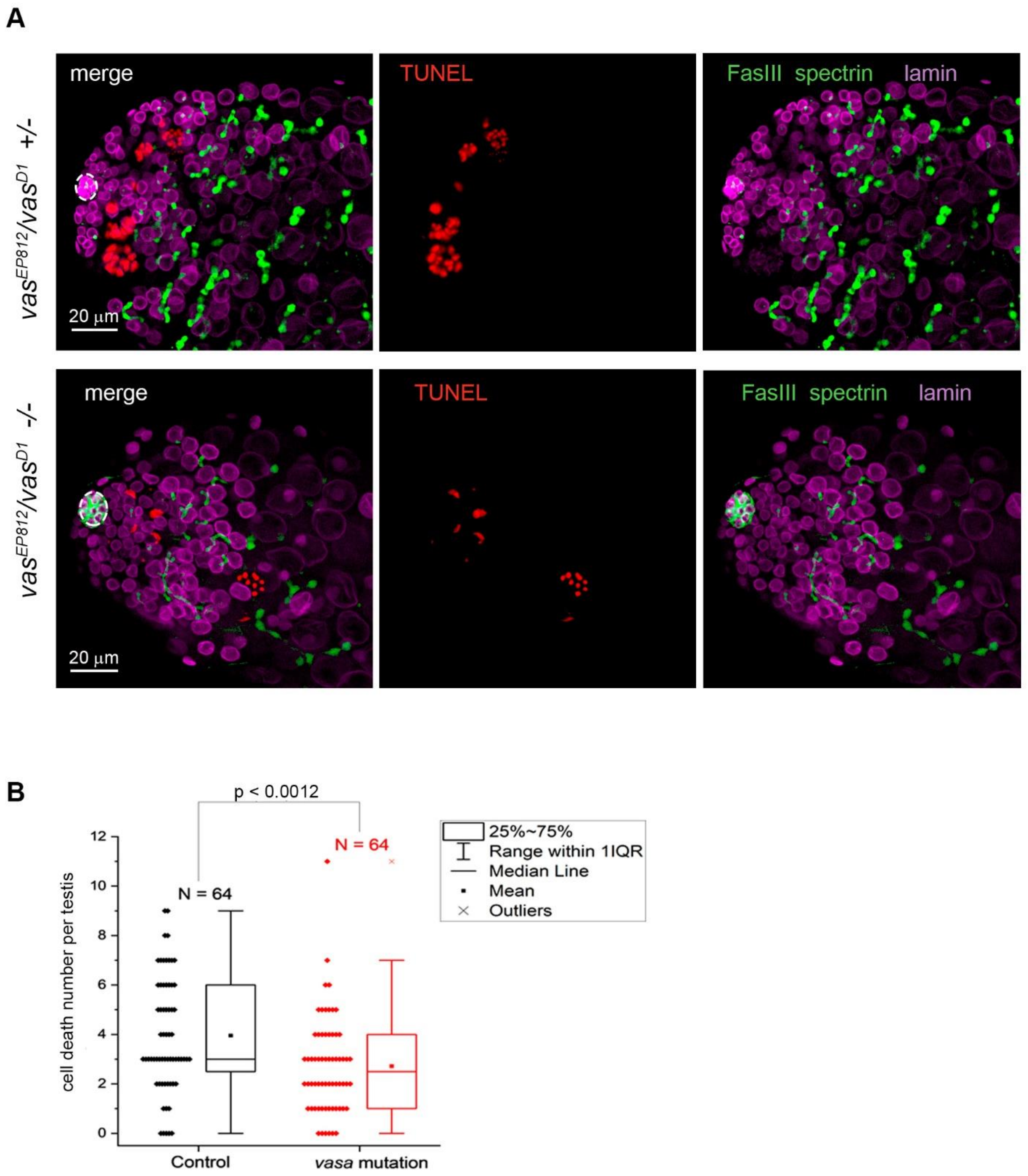

Figure S2. Analysis of programmed cell death events in *vasa* mutant and control testes. (**A**) TUNEL assay (red signals) with simultaneous immunostaining with anti-Fasciclin III (FasIII, hub marker, green), α-spectrin (marker spectrosomes and fusomes, green), and anti-lamin (violet) antibodies. Confocal slices with the apical tip of the testes oriented leftward are shown. The hubs are marked by white outlines. (**B**) The data are presented as box-plot graphs of counted TUNEL-positive signals per testis. Synchronous TUNEL signals within the same cyst were considered a single event. The Wilcoxon/Mann-Whitney (U) test was used for pairwise comparison of mutant and control testes, as indicated at the top of the plot.

Figure S3

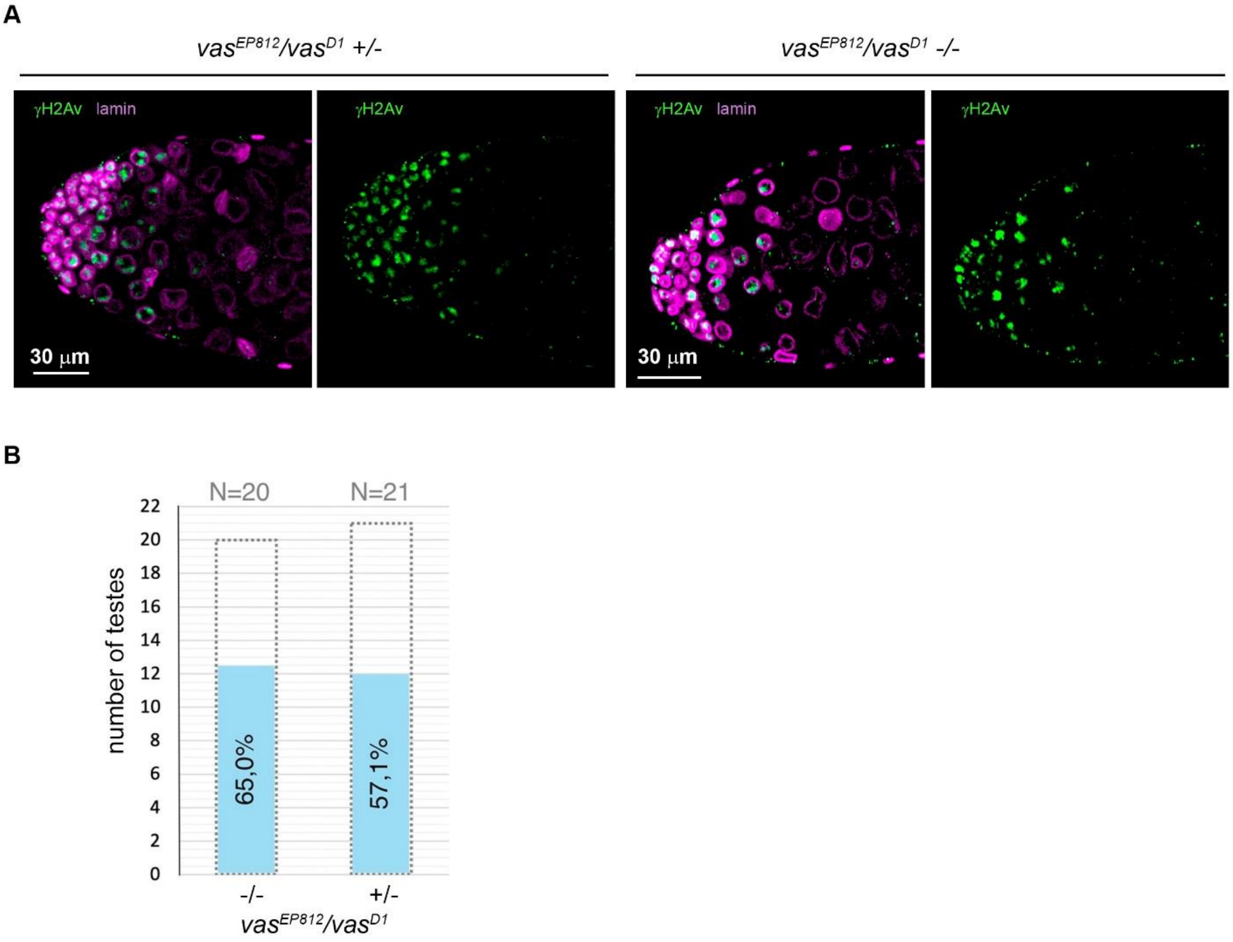

Figure S3. Analysis of double-strand breaks (DSBs) in *vasa* mutant testes. (**A**) Images of the apical tips of the testes of *vasa* mutant and heterozygous control males (*vas*^{EP812}/*vas*^{D1}-/- and +/-), obtained by confocal microscopy of immunostained testes preparations with antibodies to lamin (a marker of nuclear envelope, violet) and γ-H2aV (a marker of DNA DSBs, green). Accumulation of DNA double-strand breaks (DSBs) in male early germ cells of *vasa* mutant and control males (0 day after eclosion) is similar. (**B**) Distribution of the testes of *vas*^{EP812}/*vas*^{D1} mutant and heterozygous control males with γ-H2Av-positive signals (blue bars) and γ-H2Av-negative ones (bars without color). Data are represented as percentages of γ-H2Av-positive testes. N, the number of analyzed experimental and control testes.

Figure S4

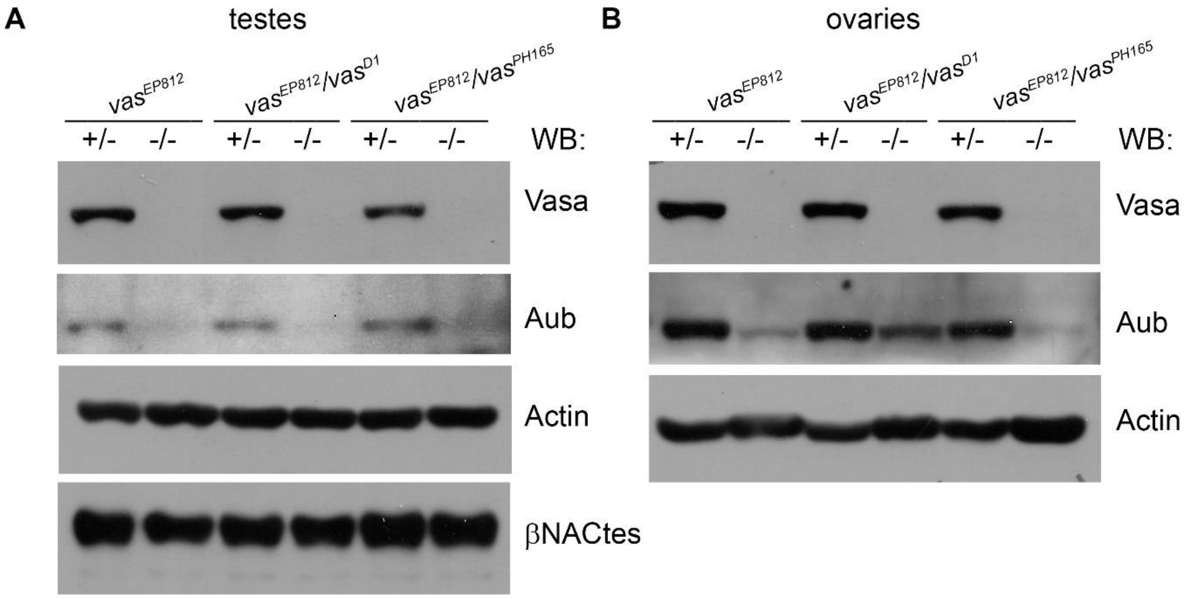

Figure S4. Western blot analysis of testis (**A**) and ovarian (**B**) lysates of *vasa* mutants and heterozygous controls with antibodies to Aub, Vasa, Actin, and βNACtes (last only for testis lysates). Antibodies to βNACtes (a marker of spermatocytes) were used for control of total germline content in testes. Anti-Actin antibodies were used as a loading control. Representative results from at least four independent experiments are presented. Testes of 0-day-old males and ovaries of females (3-5 days after eclosion) were used for lysate preparations.

Figure S5

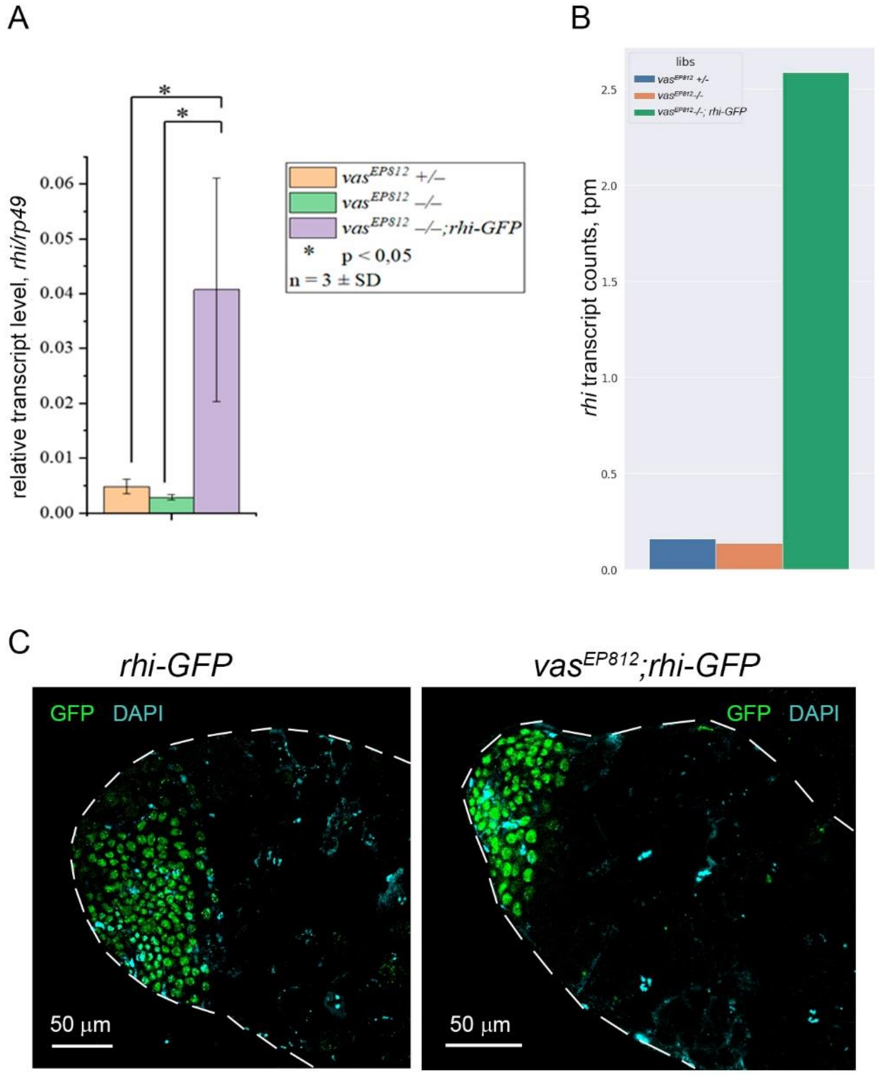

Figure S5. (**A**) RT-qPCR analysis of *rhi* transcript level in heterozygous *vas*^{EP812}+/-control testes, mutant *vas*^{EP812}-/- testes, and *vas*^{EP812}-/-; *rhi-GFP* testes. *Rhi-GFP* construct is found to be highly transcribed in the background of *vasa* mutation. (**B**) Counts of *rhi* transcript reads in tpm in RNA-seq libraries from the testes of *vas*^{EP812}+/-, *vasEP812-/-*, and *vas*^{EP812}-/-; *rhi-GFP* males. See Materials and Methods for details of the analysis. (**C**) Images of the testis tips of *rhino-GFP* and *vas*^{EP812}-/-;*rhino-GFP* males obtained by confocal microscopy of unfixed testis preparations with DAPI staining (blue) and endogenous GFP signals (green) with an excitation light wavelength of 488 nm. Expression of the GFP-tagged Rhi transgene is seen at the apical tips of testes.

Figure S6

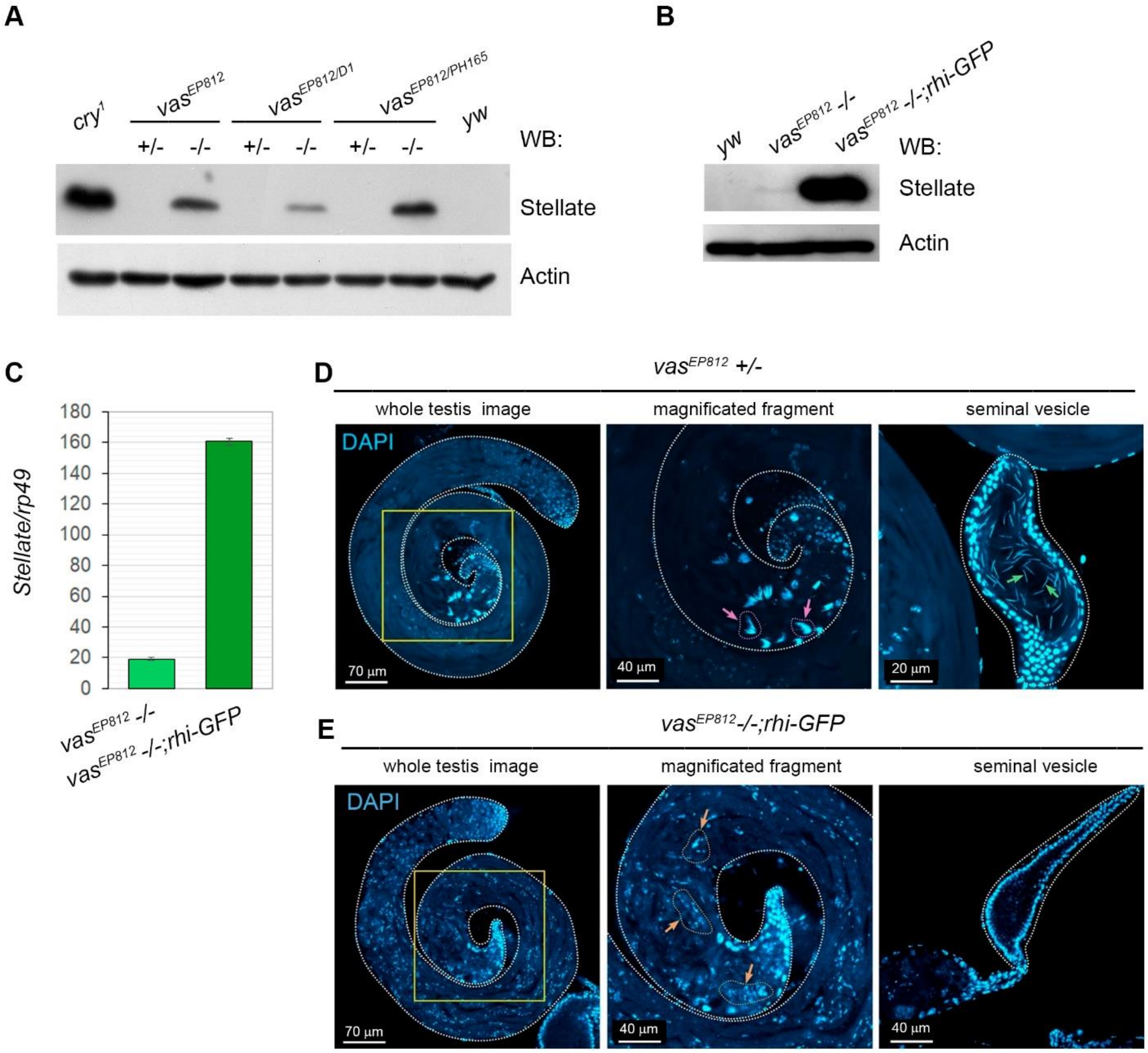

Figure S6. (**A, B**) Western blot analysis of testis lysates with anti-Stellate antibodies. Anti-Actin antibodies were used as a loading control. The testes of cry^{1} line with the depletion of the *Su(Ste)* locus were used as a positive control for *Stellate* derepression. The testes of *yw* males were used as wild-type control. (**C**) Estimation of *Stellate* copy number in the genome. The results of qPCR with genomic DNA for the testes vas^{EP812}*-/-* and vas^{EP812}*-/-;rhi-GFP* using *Stellate*-specific primers with *rp49* normalization. The number of *Stellates* in the genomes of indicated fly lines is shown on the *y* axis. (**D**) Analysis of postmeiotic spermatogenesis stages in the testes of vas^{EP812}*+/-* males. The testis preparations were stained with DAPI (blue). Left image: whole testis. Middle image: magnified fragment of the left image indicated by the yellow box. Violet arrows indicate the heads of elongated spermatid bundles. Right image: the seminal vesicle filled with mature sperm (green arrows). (**E**) Analysis of postmeiotic spermatogenesis stages in the testes of vas^{EP812}*-/-;rhi-GFP* males. The testis preparations were stained with DAPI (blue). Left image: whole testis. Middle image: magnified fragment of the left image indicated by the yellow box. Red arrows indicate heads of elongated spermatid with the disrupted individualization process. Right image: the seminal vesicle without sperm.
